# *Caligus rogercresseyi* acetylcholinesterase types and variants: a potential marker for organophosphate resistance

**DOI:** 10.1186/s13071-018-3151-7

**Published:** 2018-10-30

**Authors:** Celia Agusti-Ridaura, Michael Dondrup, Tor E. Horsberg, Jong S. Leong, Ben F. Koop, Sandra Bravo, Julio Mendoza, Kiranpreet Kaur

**Affiliations:** 1 Faculty of Veterinary Medicine, Norwegian University of Life Sciences, Sea Lice Research Centre, Postboks 369 Sentrum, Oslo, NO-0102 Norway; 2 Department of Informatics, University of Bergen, Sea Lice Research Centre, Thormøhlensgate 55, N-5008 Bergen, Norway; 3 Biology Department, Centre for Biomedical Research, University of Victoria, Station CSC, PO Box 1700, Victoria, BC V8W 2Y2 Canada; 4 Universidad Austral de Chile, Casilla 1327, Puerto Montt, Chile; 5 Cermaq Chile, Diego Portales 2000, Puerto Montt, Chile

**Keywords:** *Caligus rogercresseyi*, Acetylcholinesterase, Organophosphate, Azamethiphos, Resistance, Variants, Mutation

## Abstract

**Background:**

Control of the sea louse *Caligus rogercresseyi* in the Chilean salmonid industry is reliant on chemical treatments. Azamethiphos was introduced in 2013, although other organophosphates were previously used. In 2014, reduced sensitivity to azamethiphos was detected in the Los Lagos Region using bioassays. The main target of organophosphates is the enzyme acetylcholinesterase (AChE). Mutations in the AChE gene are the main cause of organophosphate resistance in arthropods, including other sea lice. In the present study, we aimed to characterize *C. rogercresseyi* AChE(s) gene(s) and to study the association between AChE variants and azamethiphos resistance in this sea louse species.

**Methods:**

Samples of adult male and female *C. rogercresseyi* were collected in the Los Lagos Region in 2014. Twenty-four hour exposure bioassays with azamethiphos were performed to select sensitive and resistant lice. The full-length cDNA coding sequences encoding for two AChEs in *C. rogercresseyi* were molecularly characterized. One of the AChE genes was screened by direct sequencing in the azamethiphos-selected lice to search for variants. An additional louse sampling was performed before and after an azamethiphos treatment in the field in 2017 to validate the findings.

**Results:**

The molecular analysis revealed two putative AChEs in *C. rogercresseyi*. *In silico* analysis and 3D modelling of the protein sequences identified both of them as invertebrate AChE type 1; they were named *C. rogercresseyi* AChE1a and 1b. AChE1a had the characteristics of the main synaptic AChE, while AChE1b lacked some of the important amino acids of a typical AChE. A missense change found in the main synaptic AChE (1a), F318F/V (F290 in *Torpedo californica*), was associated with survival of *C. rogercresseyi* at high azamethiphos concentrations (bioassays and field treatment). The amino acid change was located in the acyl pocket of the active-site gorge of the protein.

**Conclusions:**

The present study demonstrates the presence of two types of AChE1 genes in *C. rogercresseyi*. Although enzymatic assays are needed, AChE1a is most probably the main synaptic AChE. The function of AChE1b is unknown, but evidence points to a scavenger role. The AChE1a F/V318 variant is most probably involved in organophosphate resistance, and can be a good marker for resistance monitoring.

**Electronic supplementary material:**

The online version of this article (10.1186/s13071-018-3151-7) contains supplementary material, which is available to authorized users.

## Background

The sea louse *Caligus rogercresseyi* (Copepoda, Caligidae) is the most harmful parasite affecting the salmon farming industry in Chile [1–3]. Heavily infested fish show physiological changes due to stress, lice-induced skin lesions, weight loss and increased susceptibility towards other infectious diseases [4]. In addition to the economic loss due to the damage inflicted to fish, salmon producers must face the additional cost of anti-lice treatments. *Caligus rogercresseyi* has a wide host range, parasitizing both wild and farmed fish (reviewed in [5]). Its direct life-cycle facilitates the transmission between hosts, especially in densely reared fish [5].

Control of *C. rogercresseyi* infestations in Chilean fish farms depends mainly on the use of anti-lice chemicals (reviewed in [6, 7]). The organophosphates metrifonate and dichlorvos (applied by bath) were used during the period 1981–2000 [8]. Between 2000 and 2007, the only anti-lice chemical authorized in Chile was the avermectin emamectin benzoate [9, 10]. Reduction in treatment efficacy by emamectin benzoate was first detected in 2006–2007 [9–11]. Pyrethroids were introduced (deltamethrin in 2007 and cypermethrin in 2010) to cope with the resistance problem of *C. rogercresseyi* towards emamectin benzoate. Pyrethroids became the main anti-lice chemical agents in use until 2013, when treatment failures due to pyrethroid-resistant parasites were reported [12]. In order to control *C. rogercresseyi* loads in salmonid farms, the organophosphate azamethiphos was introduced in 2013 [7]. Since then, azamethiphos is the most utilized anti-lice chemical in Chilean salmonid farming [13].

Sea lice resistance towards the main anti-lice chemicals has been recorded in most salmonid producing countries. Organophosphate-resistant salmon lice, *Lepeophtheirus salmonis*, have been found in Scotland, Norway and Ireland [14–17]. Reduced sensitivity of *C. rogercresseyi* towards azamethiphos has been reported recently in some salmonid production areas in Chile [7, 18]. Agusti et al. [7] showed that azamethiphos treatment efficacy for adult *C. rogercresseyi* ranged between 92 and 100% in four farms located in Los Lagos and Aysén Regions (commonly known as Regions X and XI, respectively). However, when lice sensitivity to azamethiphos was tested in Los Lagos Region by the use of bioassays, Agusti et al. [7] reported EC_50_ values (the concentration immobilizing 50% of the parasites) between eight and fifteen times higher than the *C. rogercresseyi* putative naïve level (sensitivity of lice never exposed to an anti-louse chemical). Marín et al. [18] also observed reduced sensitivity of *C. rogercresseyi* to azamethiphos in several farms in Los Lagos and Aysén Regions, and found that lice from Los Lagos Region tended to be less sensitive to azamethiphos than those from Aysén Region. Agusti et al. [7] suggested the previous (1981–2001) and present (2013–2016) use of organophosphates as the possible cause for the reduced sensitivity observed in Los Lagos Region.

Due to the incipient resistance development in *C. rogercresseyi* to azamethiphos, an accurate time-space monitoring of the sensitivity level to this chemical is highly necessary. Currently, bioassays are the only available method for measuring *C. rogercresseyi* sensitivity in Chile [7, 12, 18]. In these assays, live sea lice are exposed to a range of chemical concentrations for a given time. Following the exposure, the number of dead or immobilized parasites is registered [19]. However, bioassays are laborious to conduct and subject to several potential methodological and interpretation errors (discussed in [20, 21]). Molecular methods have demonstrated to be a powerful tool for monitoring the sensitivity of sea lice to chemicals [22, 23]. However, it is important to elucidate the molecular mechanisms behind the resistance for developing such a tool.

Mutations in the gene coding for the enzyme acetylcholinesterase (AChE) have been reported as the main cause of resistance towards organophosphates in arthropods, followed by metabolic and behavioural mechanisms (discussed in [21, 24–28]). AChE is present in the cholinergic synapses of the nervous system of arthropods and in the neuromuscular junctions of vertebrates [29]. It hydrolyses the neurotransmitter acetylcholine, assuring the correct functioning of neurotransmission. Organophosphates inhibit AChE, causing the accumulation of acetylcholine in the synapses (reviewed in [24, 25, 28, 30]). This results in overstimulation of nerves and muscles, leading to paralysis and death of the parasite. Kaur et al. [21, 22] found that a single amino acid mutation (F362Y, corresponding to Phe331 in *Torpedo californica* AChE) in the active-site gorge of the synaptic AChE was the major factor responsible for organophosphate resistance in *L. salmonis*. Based on those findings, a rapid and reliable assay was developed for screening the mutation in field samples, providing a valuable tool for salmon louse resistance monitoring [21, 22].

The main aim of the present study was to identify and characterize the AChEs present in adult *C. rogercresseyi* collected in Chilean salmonid farms. In order to explore the possible causes for the loss of sensitivity to azamethiphos in some louse populations, the gene coding for the synaptic AChE enzyme (*C. rogercresseyi* AChE1a) was screened for variants in sensitive and resistant lice.

## Methods

### Sea lice sampling and selection

**Fig. 1:**
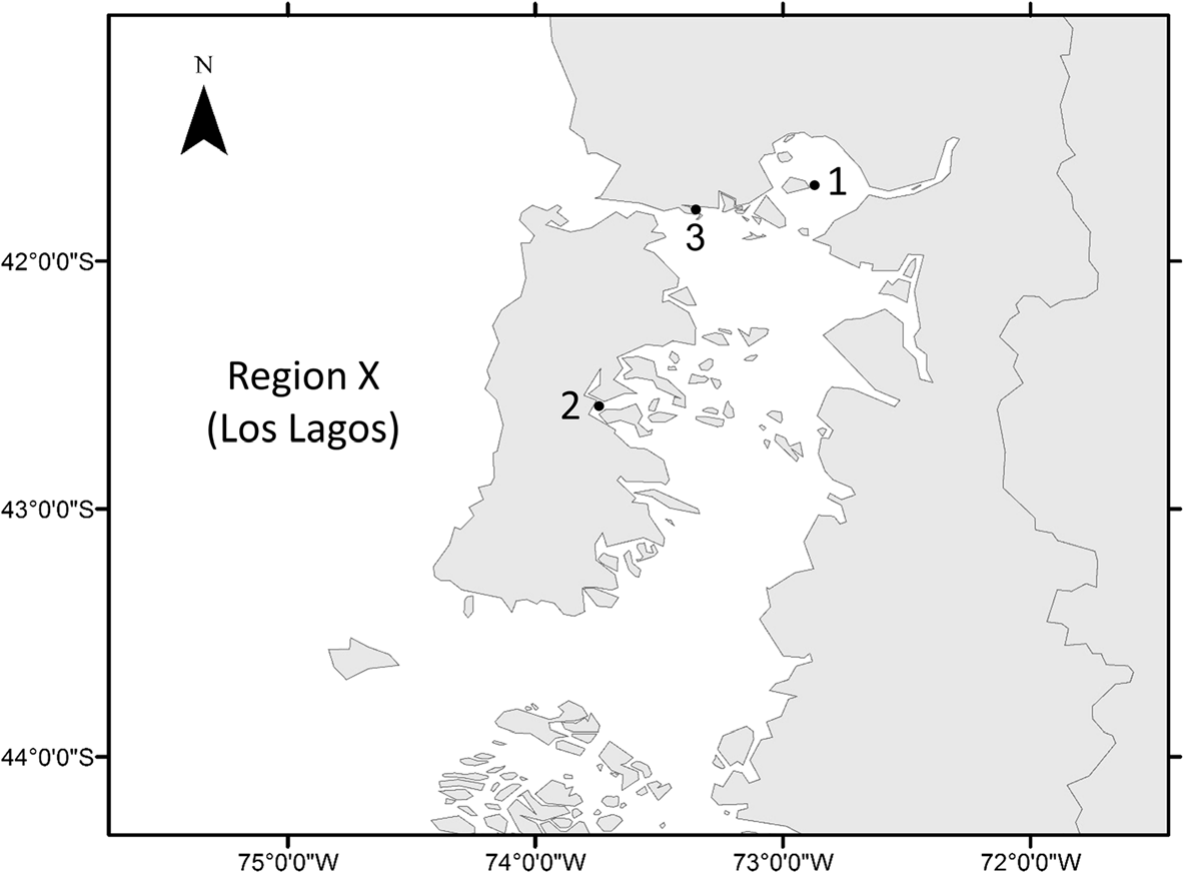
Farm locations where *Caligus rogercresseyi* were collected in March 2014 (Farms 1 and 2) and in June 2017 (Farm 3). Map displays the interior sea in Los Lagos Region (Region X)

*Caligus rogercresseyi* individuals were collected at two-time points: during March 2014 and June 2017. Three sampling sites were chosen based on the availability of lice in farms in Los Lagos Region and the suspected different lice sensitivities to azamethiphos [7, 18]. The number of treatments applied in each farm and the treatment efficacies were used as an indicator for farm selection, since low lice sensitivity seems to be related with high number of treatments and low treatment efficacies [7, 23]. The farms selected were located in the north and middle part of the interior sea in Los Lagos Region (Fig. 1). During the year before the lice were collected, Farm 1 conducted two azamethiphos treatments and Farm 2 seven treatments before March 2014; Farm 3 conducted four azamethiphos treatments before lice sampling in June 2017. All farms were stocked with a single year-class of fish. Farms 1 and 3 reared Atlantic salmon and Farm 2 reared rainbow trout. Fish were sampled using a dip net and anesthetized for louse collection.

Bioassays were conducted on lice collected in Farms 1 and 2 during March 2014 in order to assess the actual lice sensitivity to azamethiphos and for a proper selection of sensitive *versus* resistant lice. Lice used for bioassays were all adult males and females, but differences in the maturation level of the genital segment were observed. Bioassays were carried out as described by Agusti et al. [7], Helgesen et al. [12] and Helgesen & Horsberg [19]. *Caligus rogercresseyi* were gently removed from the anesthetized fish using forceps. They were kept alive at low temperature in containers with seawater supplied with aeration until the start of the bioassay. Sea lice were exposed to azamethiphos for 24 h at 12 °C with constant aeration, and the results were recorded immediately following the exposure time. Two types of bioassays were designed depending on the number of azamethiphos dilutions used and the purpose of the bioassay. For sensitivity assessment (and lice selection), four to five azamethiphos dilutions were prepared in filtered (100 μm filter) seawater, ranging from 0.2 to 2 ppb (μg l^-1^). For lice selection only, two azamethiphos dilutions were used: 0.4 and 2 ppb. Lice immobilized (unable to attach to the walls of the containers or swim actively) at 0.4 ppb or below, were considered sensitive, while lice unaffected at 2 ppb were considered resistant [7, 19, 22, 23]. Immediately following the 24 h exposure, lice were fixed in RNAlater® (Sigma-Merck KGaA, Darmstadt, Germany; item number R0901) and kept at -80 °C. Control groups without azamethiphos were prepared to determine the general performance of the lice. Bioassays were initiated within 2–6 h of sampling the first louse. Data from four to five dose bioassays were modelled using probit modelling in JMP software, and EC_50_ values with 95% confidence intervals were calculated separately for males and females.

Fifty-seven additional lice were collected in June 2017 before and after an azamethiphos treatment event in Farm 3. Pre-treatment louse sampling was performed one day before the treatment. Thirty adult lice (12 males and 18 females) were collected from five fish during the pre-treatment sampling. The azamethiphos treatment was applied by bath at a concentration of 0.1 g/m^3^ for 50 min. The post-treatment sampling occurred three days after treatment, lice being collected from the same cage as the pre-treatment sampling. Twenty-eight adult lice (20 males and 8 females) were recovered after treatment from 20 fish. Lice were gently removed from the anesthetized fish using forceps and were transferred directly to RNAlater® for fixation and preservation.

### RNA extraction and cDNA synthesis

Total RNA was extracted from the whole body (lice from Farms 1 and 2; bioassays) or only from the cephalotorax (lice from Farm 3; pre-post azamethiphos treatment) of adult *C. rogercresseyi* females and males. A Trizol protocol combined with the RNeasy Mini kit for animal tissues (Qiagen, Hilden, Germany) was used for the extraction. Lice tissues were homogenized in 1 ml Trizol using TissueLyser MM 301 (Qiagen Retsch, Hilden, Germany) and one stainless steel bead of 5 mm diameter (Qiagen). After mixing with 0.2 ml chloroform and a centrifugation step, the aqueous phase was transferred to a new vial and mixed with one volume of 70% ethanol. Total RNA was then isolated with RNeasy spin columns following the manufacturer’s protocol. The RNA was quantified with an ND-100 Spectrophotometer (Thermo Fisher Scientific, Waltham, Massachusetts, USA) and the quality was checked with a 2100 Bioanalyzer instrument (Agilent Technologies, Santa Clara, California, USA) and an Agilent RNA 6000 Nano kit. First strand cDNA was synthesized with the RevertAid H Minus first strand cDNA synthesis kit (Thermo Fisher Scientific) using oligo (dT)_18_ primers.

### Identification and characterization of *C. rogercresseyi* AChEs

In order to identify *C. rogercresseyi* AChE sequences, a search against the *C. rogercresseyi* transcriptome [31] was conducted using the two types of AChEs in *L. salmonis* (*ace1a* and *ace1b*) as queries. The search was performed using blastn optimized for discontiguous megablast [32]. To verify and properly characterize the *C. rogercresseyi* sequences obtained, the cDNA sequences were used for searching against the *C. rogercresseyi* Whole-Genome Shotgun Contigs (WGS) [33] (blastn, megablast, NCBI [32]). The cDNA and genomic sequences were assembled using ContigExpress software (Vector NTI Advance 11.0, Invitrogen Corp. 2008). The sequences obtained were amplified and analyzed on a subset of lice enrolled in the bioassay analysis (*n* = 10). The PCR cycling conditions were as follows: 98 °C for 2 min followed by 35 cycles of amplification at 98 °C for 10 s, 62 °C or 67 °C for 15 s (see Additional file 1 for primer annealing temperature), 72 °C for 2 min and a final extension step at 72 °C for 5 min. The primers selected were located in the putative untranslated 5' and 3' regions (UTRs) and are listed in Additional file 1. The Phusion® High-Fidelity DNA Polymerase (New England BioLabs, Ipswich, Massachusetts, USA) was utilized. Amplicons were subjected to direct sequencing using several internal primers (Additional file 1) (GATC Biotech, Constance, Germany; Sanger sequencing service, Supremerun sequencing).

*In silico* analysis of the deduced *C. rogercresseyi* AChE protein sequences were performed as follows. Database searches of the sequences were conducted against several databases in order to verify the identity of the proteins: GenBank (non-redundant protein sequences - nr, BlastP), UniProtKB (BlastP; default settings) and InterProScan sequence search (default settings). In addition, the deduced proteins were compared to previously published AChE protein sequences from another 19 species, using the ClustalO program [34] with default settings to obtain a multiple sequence alignment (MSA). The following online modelling platforms were used for predicting the presence of transmembrane helices in *C. rogercresseyi* AChEs: SOSUI [35, 36], TMHMM Server v.2.0 [37–39] and DAS [40, 41].

A phylogenetic analysis of the putative *C. rogercresseyi* AChEs was performed. Several cholinesterase (ChE) types from vertebrates and invertebrates were included. Full-length ChE sequences were found by: (i) BlastP searches of the GenBank-nr and UniprotKB databases using the *C. rogercresseyi* AChE sequences identified in this study as queries; and (ii) searching for complete sequences in the UniprotKB database, using keywords as queries (“acetylcholinesterase”, “cholinesterase”, “ace”, “AChE”, etc.). MSA was generated with MUSCLE [42] with default parameters. MSA was manually inspected and edited in Jalview [43] removing regions of low coverage, low alignment quality, and a complete sequence introducing large gaps (*Gallus gallus* AChE). Phylogenetic trees were constructed using MrBayes v.3.2.6 x64 [44] using 8 parallel chains over a total of 5 million generations; rates were approximated by a Γ-distribution with 4 categories, allowing for invariant sites. Markov Chain Monte Carlo (MCMC) analysis was run in mixed mode [45] to allow MrBayes to determine the optimal substitution matrix, resulting in a WAG+I+Γ model. In addition, the MSA was submitted to ProtTest server [46] to determine the optimal substitution model and MrBayes was run again using the best-fit LG+I+Γ model. The vertebrate taxa were used as out-group to root the trees.

To further explore the identity of *C. rogercresseyi* AChE sequences, another phylogenetic analysis was performed with the same parameters. The analysis included sequences of several invertebrate and vertebrate carboxylesterases, esterases and other cholinesterases available in the UniprotKB database.

### 3D modelling of *C. rogercresseyi* AChEs and docking analysis

The three-dimensional (3D) structure of the *C. rogercresseyi* AChEs was modelled using SWISS MODEL in the automated mode [47–49]. For a better search of templates, a number of amino acids at each end of the *C. rogercresseyi* AChEs sequences were truncated following a similar pattern as in *Drosophila melanogaster* and *Homo sapiens* AChE [Protein Data Bank (PDB) codes 1qo9.1 and 4ey4.1, respectively]. In short, the AChEs sequences were aligned using ClustalO program with default settings. The amino acids at the beginning and/or the end of the *C. rogercresseyi* AChEs that did not align to the other sequences were truncated. QMEAN and GMQE (global model quality estimation) scores were used as indicators of the quality of the models [47]. To analyze the fit between the selected templates and the predicted *C. rogercresseyi* AChEs structures, the root mean square (RMS) values for carbon alpha (Cα) were calculated using the Swiss PDB viewer v.4.1.0 [50]. The values were calculated for the whole protein and for the ten amino acids important for the function of the protein (the anionic choline binding site, the catalytic triad, the acyl pocket residues and the oxyanion hole). The numbering of amino acids is by convention from the *T. californica* AChE sequence. The 3D models were visualized using UCSF Chimera v.1.10.2. software [51]. The distances between amino acids in the active-site gorge were calculated using the same software. The volume of the active-site gorge of the proteins was calculated using the software CASTp [52, 53].

Acetylcholine and two organophosphates, azamethiphos and dichlorvos, were docked to the *C. rogercresseyi* AChEs 3D models using the online molecular docking server [54, 55]. The whole active-site gorge of the proteins, from the mouth to the bottom of the gorge, was selected for performing the analysis. The results were examined for the likely binding sites, frequency of binding to these sites, free binding energy and the estimated inhibition constant (Ki, the concentration required to produce half-maximum inhibition).

### Screening of *C. rogercresseyi ace1a* for variants

The full cDNA coding sequence of the putative synaptic AChE (1a) was amplified to look for variants, using gene specific primers (Additional file 1, “Cr_ace1a”). The sequence was analyzed in five azamethiphos sensitive, three with reduced sensitivity and two resistant adult *C. rogercresseyi* selected with bioassays (Farms 1 and 2). PCR reactions were performed using Phusion® High-Fidelity DNA Polymerase; PCR conditions and direct sequencing were performed as stated in the previous section “Methods. Identification and characterization of *C. rogercresseyi* AChEs”.

For rapid screening of the AChE1a variants in the 57 additional lice collected before and after the azamethiphos field treatment (Farm 3, June 2017), a 484 bp fragment of the *ace1a* gene containing an important missense change was amplified. Two fragment-specific primers were designed (Additional file 1, named HS4F and HS6R). PCR reactions were performed using Phusion® High-Fidelity DNA Polymerase under the following conditions: 98 °C for 2 min followed by 35 cycles of amplification at 98 °C for 10 s, 62 °C for 20 s, 72 °C for 30 s and a final extension step at 72 °C for 5 min. Amplicons were subjected to direct sequencing (GATC Biotech; Sanger sequencing service, Supremerun sequencing).

## Results

### Sequence analysis and characterization of *C. rogercresseyi* AChEs

**Fig. 2:**
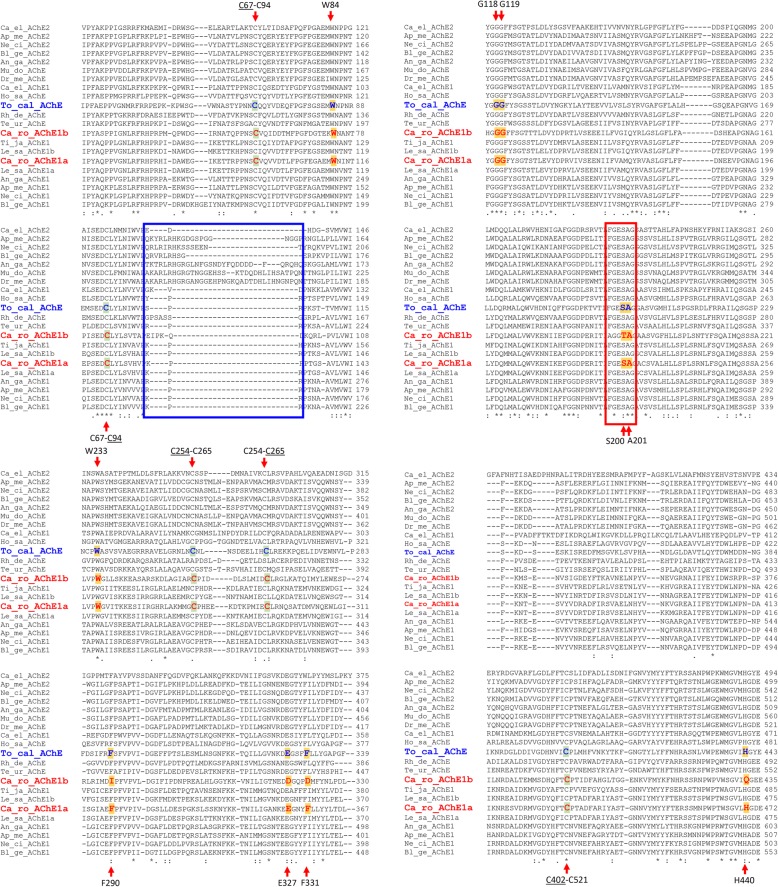
Alignment of the deduced amino acid sequence of *Caligus rogercresseyi* AChE1a and AChE1b with 19 previously published acetylcholinesterases (AChE) from other arthropods and vertebrates. The ten important amino acids and several cysteines involved in disulphide bridges are indicated by arrows (also highlighted in bold in *Torpedo californica* and *Caligus rogercresseyi* sequences, in blue and red, respectively). The red box represents the canonical “FGESAG” motif, characteristic of the active site of cholinesterases. The blue box represents the typical sequence insertion/deletion domain that distinguishes AChE1 and 2. *Abbrevations*: An_ga, *Anopheles gambiae*; Ap_me, *Apis mellifera*; Bl_ge, *Blattella germanica*; Ca_el, *Caenorhabditis elegans*; Ca_ro, *Caligus rogercresseyi*; Dr_me, *Drosophila melanogaster*; Ho_sa, *Homo sapiens*; Le_sa, *Lepeophtheirus salmonis*; Mu_do, *Musca domestica*; Ne_ci, *Nephotettix cincticeps*; Rh_de, *Rhipicephalus decoloratus*; Te_ur, *Tetranychus urticae*; Ti_ja, *Tigriopus japonicus*; To_cal, *Torpedo californica*. For UniprotKB database entry names see Additional file 3

**Fig. 3:**
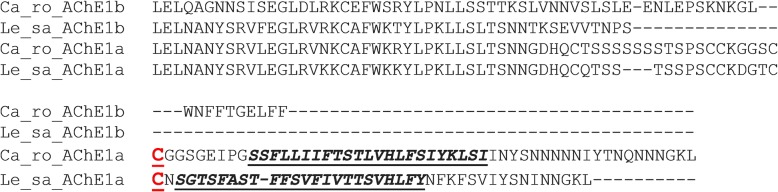
Alignment of *Caligus rogercresseyi* (Ca_ro) and *Lepeophtheirus salmonis* (Le_sa) C-terminus of AChE1a and AChE1b. The site of cleavage (cysteine) of the putative hydrophobic peptide (C595 in both louse species) is shown in bold, red and underlined. The hydrophobic C-terminal peptide in both species is in italics, bold and underlined

Two partial cDNA AChE sequences were identified in *C. rogercresseyi* transcriptome ([31] GenBank: GAZX01027370.1 and GAZX01029466.1). The search of these sequences against the *C. rogercresseyi* WGS (female and male) resulted in several contigs (see Additional file 2). The cDNA sequence of GAZX01027370.1, identified in the *C. rogercresseyi* transcriptome as a putative AChE, was confirmed and characterized in the lice collected in the present study (*n* = 10). It has an open reading frame (ORF) of 1941 bp, encoding a deduced protein of 646 amino acids. *In silico* analysis of this deduced protein identified it as an AChE with transmembrane segment. The domains and signatures identified were: cholinesterase, alpha/beta hydrolase fold, carboxylesterase type B-active site, cytoplasmic region, non-cytoplasmic region, transmembrane region and several signal peptides. The amino acid alignment of this *C. rogercresseyi* putative AChE with 19 AChE sequences from other species (Crustacea, Insecta, Nematoda, Arachnida and Vertebrata) revealed that it has the characteristic features of the main synaptic AChE (Fig. 2 and Additional file 3) [56]. These features include: the anionic choline binding site (W84, numbering corresponds to *T. californica* AChE), the three residues of the catalytic triad (S200, E327 and H440), the characteristic FGESAG motif surrounding the active serine, the acyl pocket residues (W233, F290 and F331), the oxyanion hole (G118, G119 and A201) and six cysteines involved in the three conserved disulphide bonds (C67-C94, C254-C265, C402-C521). Three online modeling platforms (SOSUI, TMHMM Server v.2.0 and DAS; see Methods for details) identified a hydrophobic C-terminal peptide in this *C. rogercresseyi* AChE as a transmembrane helix, between the amino acids 604 and 626 (*C. rogercresseyi* AChE numbering) (Fig. 3). The alignment of *C. rogercresseyi* AChE with *L. salmonis* AChE1a revealed a high identity between them (85.3%), while the identity percentage with *L. salmonis* AChE1b was 77.2%. This alignment also identified the putative *C. rogercresseyi* AChE hydrophobic peptide in a similar position as in *L. salmonis* AChE1a (Fig. 3), and the most probable cleavage site of the *C. rogercresseyi* AChE hydrophobic peptide in the cysteine residue C595 (also C595 in *L. salmonis*) (Fig. 3). Based on these observations, we concluded that this *C. rogercresseyi* AChE could be the main synaptic enzyme.

When the other cDNA sequence, GAZX01029466.1 (preliminarily identified as another AChE in the *C. rogercresseyi* transcriptome), is translated into protein, the putative start codon is located only 22 bp from the beginning of the sequence. We searched in the *C. rogercresseyi* WGS to obtain a longer 5' end fragment of the sequence. The search resulted in five genome contigs overlapping with the cDNA sequence (Additional file 2). The GAZX01029466.1 sequence was assembled with the genome contigs obtained in the blast analysis. The consensus sequence increased the putative 5' end by 279 additional bp. Primers were then designed in the new putative untranslated 5' region (UTR) and in the 3' region (Additional file 1, “Cr_ace1b”). The PCR product obtained using those primers revealed a transcript longer than the consensus sequence obtained from the assembling of GAZX01029466.1 and the genome contigs, since a completely new fragment of 484 bp was found. This fragment comprises 440 bp belonging to the 5' UTR and 44 bp to the beginning of the coding sequence, containing the “atg” start codon. The new transcript has an ORF of 1689 bp, encoding a deduced protein 562 aa long. The new 5' UTR is at least 776 bp long. The sequence was submitted to the GenBank database under the accession number MF978386. *In silico* analysis of the deduced protein revealed that it probably was an AChE without transmembrane segment. The domains and signatures identified were: cholinesterase, alpha/beta hydrolase fold and carboxylesterase type B; no cytoplasmic or transmembrane regions were identified. When the putative protein sequence was aligned with 19 AChE sequences from other species, only some of the features of a typical AChE were identified (Fig. 2): it has the anionic choline binding site (W84), the oxyanion hole (G118, G119 and A201) and six cysteine residues involved in three conserved disulphide bonds (C67-C94, C254-C265, C402-C521). However, all three residues of the catalytic triad and two residues of the acyl pocket are substituted by other amino acids, and it lacks the characteristic FGESAG motif. The three online modeling platforms used for predicting the presence of transmembrane helices (SOSUI, TMHMM Server v. 2.0 and DAS), predicted this protein to be soluble, without hydrophobic peptide. In addition, no free cysteine residue (the potential cleavage site of a hydrophobic peptide) is present in the C-terminal of the protein (Fig. 3), as is the case in *L. salmonis* AChE1b. The most similar proteins identified were *L. salmonis* AChE1a (54.9%) and AChE1b (54.9%). It shares 54% similarity with the other AChE described above for *C. rogercresseyi*.

**Fig. 4:**
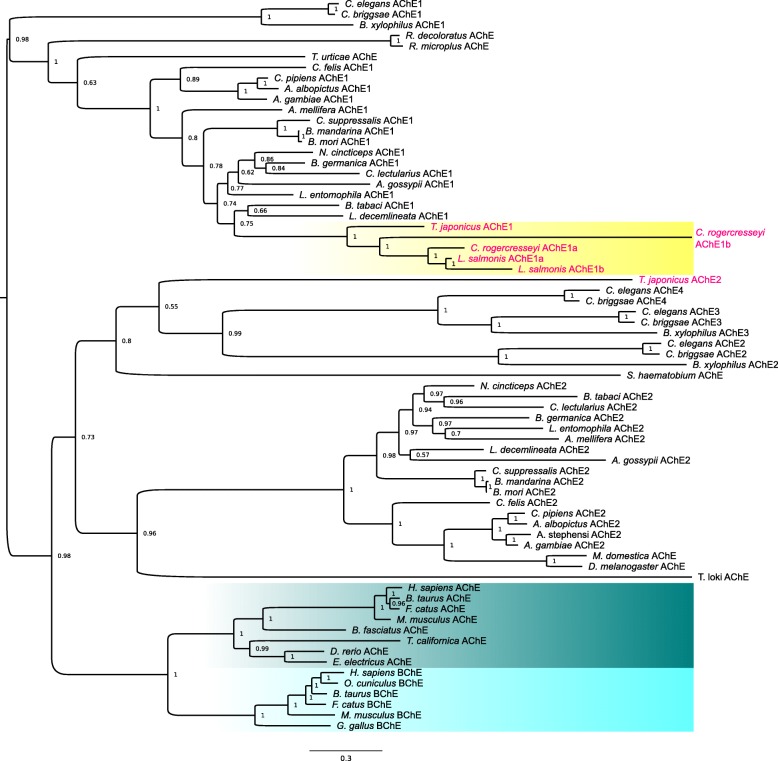
Phylogenetic analysis including *Caligus rogercresseyi* AChE1a and AChE1b together with several cholinesterases from vertebrates (blue box) and invertebrates. *Caligus rogercresseyi* AChE1a and 1b are shown in a yellow box together with other crustacean AChEs. For abbrevations and UniprotKB database entry names see Additional file 3

Phylogenetic analysis (Fig. 4 and Additional files 3 and 4) showed that the two putative *C. rogercresseyi* AChE sequences cluster together with the AChE type 1 occurring in crustaceans and insects, being clearly separated from the AChE type 2 group. Based on this finding, the protein deduced from the GAZX01027370.1 sequence will be hereafter referred to as *C. rogercresseyi* AChE1a, and the protein deduced from the new sequence generated on basis of GAZX01029466.1, as *C. rogercresseyi* AChE1b. *Caligus rogercresseyi* AChE1a grouped together with both *L. salmonis* AChEs in the phylogenetic tree. *Caligus rogercresseyi* AChE1b appeared in the crustacean AChE type 1 cluster, but with a large genetic distance. The inclusion of several invertebrate and vertebrate carboxylesterases, esterases and other cholinesterases did not change the position of *C. rogercresseyi* AChE1b in the tree (Additional file 4).

### Bioassays and field treatment with azamethiphos

Bioassays with 24 h exposure time were performed to determine the lice sensitivity towards azamethiphos, at the population and individual levels. In Farms 1 and 2, one 5/6-dose bioassay was performed per farm. The lice sensitivity level to azamethiphos, expressed as EC_50_ values with 95% confidence intervals, was 0.48 ppb (0.09–2.50) for males and 0.51 ppb (0.26–0.99) for females in Farm 1, and 1.20 ppb (1.19–1.22) for both males and females in Farm 2. In addition, a 3-dose bioassay for lice selection was conducted in Farm 2. Table 1 summarizes the immobilization data of *C. rogercresseyi* exposed to different azamethiphos concentrations per farm.

**Table 1:** Immobilization percentage (%) of *Caligus rogercresseyi* exposed to azamethiphos per farm using bioassays

Farm code	Aza dose (ppb)	Male immobilization % (*n*)	Female immobilization % (*n*)
1	0	0 (6)	0 (13)
	0.2	14 (7)	0 (12)
	0.4	17 (6)	42 (12)
	1.2	100 (6)	91 (11)
	2.0	100 (8)	100 (10)
2: Bioassay 1	0	12.5 (8)	0 (22)
	0.2	0 (11)	0 (19)
	0.4	0 (11)	0 (20)
	0.8	0 (8)	0 (22)
	1.2	42 (12)	37 (19)
	2	100 (10)	100 (21)
2: Bioassay 2	0	0 (19)	0 (20)
	0.4	0 (21)	0 (16)
	2.0	93 (15)	93 (15)

*Abbreviations*: *Aza* azamethiphos, *n*, sample size for each louse sex

An azamethiphos field treatment in Farm 3 was used for testing the association of an important missense change found in *ace1a* with *C. rogercresseyi* sensitivity loss towards azmethiphos. Male and female lice collected before the treatment were adults with the genital segment completely developed (“mature” following the terminology by González & Carvajal [5]), and some females carried egg strings. Treatment efficacy in the sampling cage was 97% for adult lice and 57% for chalimus stages. After the treatment, only ten adult males with fully developed genital segments (mature) were recovered on the fish analysed (*n* = 20), for the rest of the adult males (*n* = 10) and all the adult females (*n* = 8) the genital segments were not fully developed (“young adult lice” following the terminology by González & Carvajal [5]).

### Screening of *C. rogercresseyi* AChE1a for variants

The screening and alignment of the full cDNA coding sequence encoding for *C. rogercresseyi* AChE1a in five sensitive (immobilized at 0.4 ppb or below), three with reduced sensitivity (unaffected at 1.2 ppb) and two resistant (unaffected at 2 ppb) adult *C. rogercresseyi*, revealed several synonymous and two non-synonymous changes. Additional file 5 shows the alignment of the cDNA sequences in the ten *C. rogercresseyi* individuals. One of the two non-synonymous changes found in *ace1a* was thymine to guanine at position 952 (T952G). This change led to an amino acid change: phenylalanine (F) to valine (V) at codon 318 (*C. rogercresseyi* AChE1a numbering). Among the ten *C. rogercresseyi* individuals analyzed, the F318V change was found in the two lice unaffected at 2 ppb (resistant) and in one louse immobilized at 0.4 ppb (see Table 2 for details). These three lice carrying the change were heterozygous for the variant (F/V318). The two adult lice unaffected at 2 ppb (resistant) did not have fully developed genital segments (“young adults”).

**Table 2:** Presence/absence of the F/V318 variant in *Caligus rogercresseyi* AChE1a selected for azamethiphos using bioassays (10 lice)

Aza dose (ppb)	Bioassay output	Louse sex	Farm	AChE1a variant
0.2	Immobilized	Male	1	Homozygous F/F
0.4	Immobilized	Male	1	Homozygous F/F
0.4	Immobilized	Female	1	Homozygous F/F
**0.4**	**Immobilized**	**Female**	**1**	**Heterozygous F/V**
0.4	Immobilized	Female	1	Homozygous F/F
1.2	Unaffected	Female	2	Homozygous F/F
1.2	Unaffected	Female	2	Homozygous F/F
1.2	Unaffected	Female	2	Homozygous F/F
**2.0**	**Unaffected**	**Female (young** ^**a**^ **)**	**2**	**Heterozygous F/V**
**2.0**	**Unaffected**	**Male (young** ^**a**^ **)**	**2**	**Heterozygous F/V**

^a^Young adult: adult lice without fully developed genital segments [5]

Lice immobilized at 0.2 and 0.4 ppb were considered sensitive, lice unaffected at 1.2 ppb were considered with reduced sensitivity and lice unaffected at 2 ppb, resistant

*Abbreviation*: *Aza* azamethiphos

Lice carrying the variant are highlighted in bold

To investigate the importance of the F/V318 variant, the sequence of the *C. rogercresseyi* AChE1a protein was aligned with 19 AChE amino acid sequences from other species (Fig. 2). The alignment revealed that F318 in *C. rogercresseyi* is homologous to F290 in the *T. californica* AChE. This amino acid is located in the acyl pocket of the protein, neighboring the catalytic center in the active-site gorge. It is a highly conserved residue among the species as evident from the MSA of AChEs from several species (Fig. 2).

The other non-synonymous change found in *C. rogercresseyi ace1a*, guanine to adenine at position 82 (G82A), led to the amino acid change valine to isoleucine at position 28 (V28I). This change was found in only one sensitive male louse and it is situated close to the N-terminus of the protein. Valine and isoleucine are similar amino acids with similar physical properties. In addition, when aligning *L. salmonis* AChE1a, AChE1b and *C. rogercresseyi* AChE1a sequences, both *L. salmonis* AChEs has isoleucine at that same position (data not shown).

The analysis of the AChE1a fragment in the lice collected before and after the azamethiphos field treatment showed that every young adult female recovered after treatment carried the F/V318 variant. Table 3 shows the percentage of lice carrying the F/V318 variant in the pre- and post-treatment sampling, separate for sex and maturation level of the genital segment. All lice carrying the change were heterozygous for the variant (F/V318).

**Table 3:** Percentage (%) of *Caligus rogercresseyi* carrying the F/V318 variant sampled before and after the azamethiphos field treatment in Farm 3

	Before treatment	After treatment
Adult males	8.3 (1/12)	21.1 (4/19)
Young^a^ males	Not sampled	30 (3/10)
Mature^a^ males	8.3 (1/12)	11.1 (1/9^b^)
Adult females	50 (9/18)	100 (8/8)
Young^a^ females	Not sampled	100 (8/8)
Mature^a^ females	50 (9/18)	Not found on fish

^a^Young adults: adult lice with the genital segment not fully developed. Mature adults: adult lice with fully developed genital segment [5]

^b^Ten mature males recovered in total, but no sequence information from one male due to poor RNA quality

The number of lice harboring the variant out of the total number of lice per group is shown in parentheses

### 3D modelling of *C. rogercresseyi* AChEs and docking analysis

The 3D structure of *C. rogercresseyi* AChE1a and AChE1b was modeled using the SWISS MODEL software. An initial template search revealed several possible templates. The 3D model giving high QMEAN and GMQE scores in the template search for *C. rogercresseyi* AChE1a was the crystal structure of recombinant human AChE in the apo state (PDB code 4ey4.1) (Table 4). Native *D. melanogaster* AChE (PDB code 1qo9.1) gave lower GMQE and QMEAN values than human AChE as template (Table 4). *Drosophila melanogaster* AChE is the only arthropod AChE which crystalline 3D structure has been solved to date [57]. Since *C. rogercresseyi* is also an arthropod, *D. melanogaster* AChE is expected to be a relatively good template for predicting the 3D structure of *C. rogercresseyi* AChE1a. However, they belong to different AChE types, *D. melanogaster* AChE belonging to type 2 and *C. rogercresseyi* AChE to type 1 (see Fig. 4), which could explain the low GMQE and QMEAN values. The RSM values (Cα) for recombinant human AChE, the native *D. melanogaster* AChE and the predicted structure of *C. rogercresseyi* AChE1a (F318 and V318 variants) are shown in Table 4. Since the model quality indicators and fit values were better for human AChE as template for the two *C. rogercresseyi* AChE1a variants than *D. melanogaster* AChE, human AChE 3D structure was chosen to predict both variants of *C. rogercresseyi* AChE1a. For *C. rogercresseyi* AChE1b, the crystal structure of fully glycosylated human butyrylcholinesterase (BChE) was chosen (PDB code 4aqd.1) (see Table 4 for GMQE, QMEAN and RSM values). Additional files 6, 7 and 8 contain the PDB files of the predicted *C. rogercresseyi* AChE1a (F318 and V318 variants) and AChE1b 3D structures.

**Table 4:** 3D modelling of *Caligus rogercresseyi* AChE1a (F318 and V318 variants) and AChE1b

Template	Cr AChE1a (F318)		Cr AChE1a (V318)		Cr AChE1b
	Hs AChE (4ey4.1)	Dm AChE (1qo9.1)	Hs AChE (4ey4.1)	Dm AChE (1qo9.1)	Hs BChE (4aqd.1)
GMQE	0.77	0.74	0.77	0.74	0.74
QMEAN	-1.66	-3.91	-1.66	-3.91	-2.87
RSM wp (Å)	0.28	0.35	0.28	0.39	0.26
RSM 10 aa (Å)	0.13	0.34	0.13	0.23	0.25

*Abbreviations*: *Cr Caligus rogercresseyi*, *Hs AChE* recombinant human AChE in the apo state (PDB codes are shown in parentheses), *Dm AChE* native *Drosophila melanogaster* AChE, *Hs BChE* fully glycosylated human butyrylcholinesterase, *QMEAN* and *GMQE* scores for the model quality, *RMS (in Å)* fit between template and model (wp: whole protein; 10 aa: ten important amino acids)

**Fig. 5:**
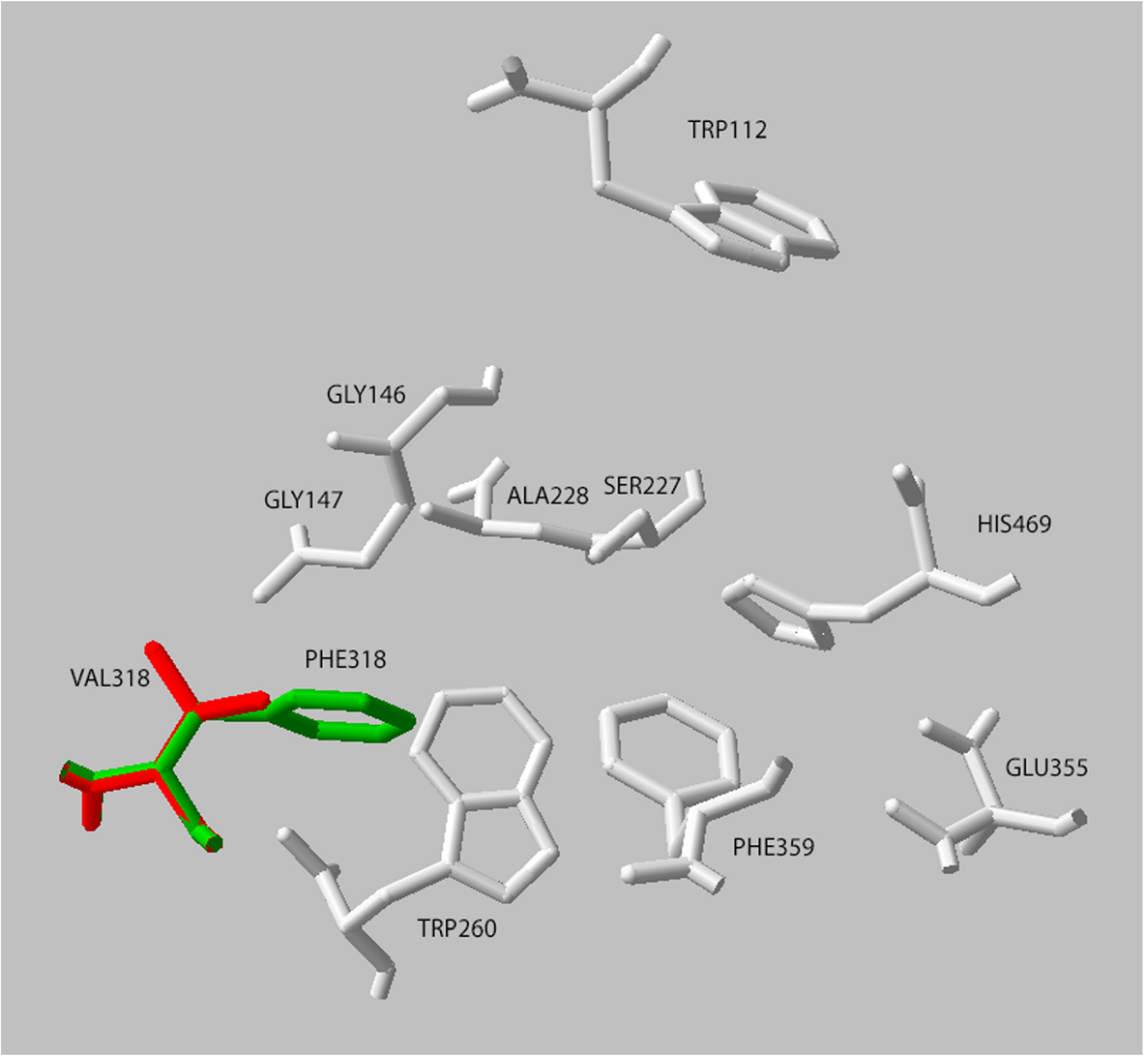
Overlay of the predicted 3D positioning of the ten functionally important amino acids in both *Caligus rogercresseyi* AChE1a variants. Numbering corresponds to *Caligus rogercresseyi* AChE1a. The phenylalanine at position 318 (F290, *Torpedo californica* numbering) is displayed in green, while the valine at that same position is in red

**Fig. 6:**
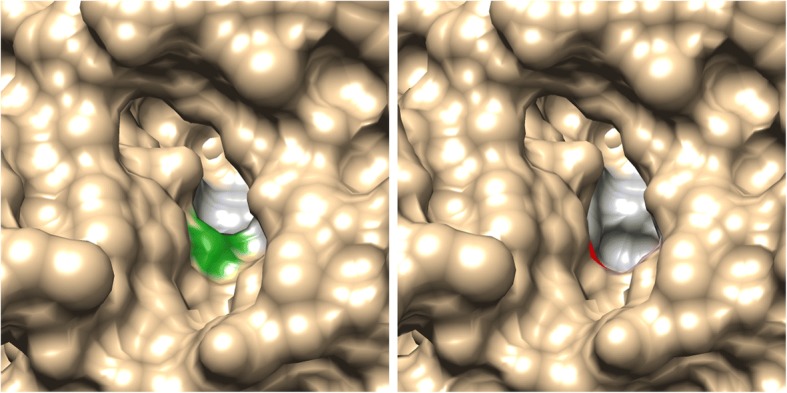
Surface of the predicted 3D structure of *Caligus rogercresseyi* AChE1a F318 variant (left panel, displayed on green) and V318 (right panel, displayed on red). View from the mouth of the active-site gorge of the protein

The 3D modeling of *C. rogercresseyi* AChE1a predicted a gorge containing the ten amino acids important for the function of the protein. These amino acids were predicted in a similar position and orientation as the corresponding amino acids in the *D. melanogaster* and *H. sapiens* AChE templates. The predicted gorge of *C. rogercresseyi* AChE1a was about 16 Å deep and 15 Å at its widest point, close to the bottom. The entire active-site gorge had a volume similar to the gorge of the human AChE. The *C. rogercresseyi* AChE1a 3D model revealed that the change from phenylalanine to valine at position 318 resulted in a wider active-site gorge (Figs. 5 and 6): the gorge of the V318 variant is 8% bigger than the gorge of the F318 variant. Phenylalanine and valine have similar physical properties: both are nonpolar, hydrophobic and have neutral charge, but valine lacks the bulky aromatic ring of phenylalanine. The docking analysis predicted that acetylcholine binds in the active-site gorge of the F318 variant of *C. rogercresseyi* AChE1a in the proper place and orientation [56]. Acetylcholine was also predicted to bind to the active site of the V318 AChE1a variant. The organophosphates azamethiphos and dichlorvos were both predicted to bind at the mouth of the active-site gorge in the F318 variant. For azamethiphos in the V318 variant, the binding was predicted at both the mouth and the bottom of the gorge, and for dichlorvos, only at the mouth of the gorge.

The *C. rogercresseyi* AChE1b 3D model revealed a main gorge in the center of the protein, as in other AChEs and BChEs. The gorge is about 15 Å deep and 15 Å at its widest point, and its entire volume is 33% bigger than the gorge in *C. rogercresseyi* AChE1a F318 variant. *Caligus rogercresseyi* AChE1b active-site gorge is 39% smaller than human BChE. As stated above, the alignment of AChE1b and AChE1a sequences showed that some of the ten important residues that characterize a typical AChE are substituted by other amino acids in *C. rogercresseyi* AChE1b (three residues of the catalytic triad and two residues of the acyl pocket) (Fig. 2). The alignment of AChE1b and AChE1a in the 3D analysis showed that, similar to AChE1a, the substituted amino acids in AChE1b where also predicted at the bottom of the AChE1b gorge (Additional files 6 and 8). Docking analysis for AChE1b predicted that acetylcholine and azamethiphos bind inside the gorge. For dichlorvos, the analysis predicted the binding at the mouth of the gorge.

## Discussion

Decreased sensitivity of sea lice for various anti-lice chemicals has become a major issue worldwide, with Chile being no exception. Pyrethroids and emamectin benzoate have been the major chemicals used aginst *C. rogercresseyi* in Chile. However, the development of resistance over the years, attributed to their overuse, has severely affected the fish farm industry [7, 9–12]. Because of this increased resistance, the organophosphate azamethiphos was introduced in 2013 in order to improve delousing treatment efficacies in the farms. Although azamethiphos treatments provided a good delousing effect during 2013–2014 in the four farms studied by Agusti et al. [7] (above 90% treatment efficacy for adult lice), a loss of sensitivity was reported using bioassays. Unfortunately, no molecular marker is yet available to identify resistance in *C. rogercresseyi* due to the lack of knowledge on the molecular mechanisms involved in resistance towards the chemicals used. In the present study, we investigated a possible molecular mechanism of azamethiphos sensitivity loss in *C. rogercresseyi*.

In order to elucidate the molecular mechanisms for resistance, it is important to identify and characterize the target proteins. As per the existing literature, AChEs are the target proteins for organophosphates in arthropods (reviewed in [24, 25, 28]). In the present study, we identified two AChEs in *C. rogercresseyi*, AChE1a and 1b. In *C. rogercresseyi* AChE1a, the *in silico* analysis identified it as an AChE with transmembrane region, and the alignment with AChEs from other species showed that it has the characteristic features of a main synaptic AChE [56]. The AChE1a 3D model positioned the ten important amino acids for the function of the protein at the bottom of the gorge, and the docking analysis predicted acetylcholine to bind at the proper site and orientation in the active-site gorge [56]. The phylogenetic analysis revealed that AChE1a clustered together with the AChE type 1 occurring in several arthropods. These results strongly indicate that AChE1a codes for the the main synaptic AChE. *In silico* analysis of AChE1b identified it as an AChE without transmembrane region, predicting the protein to be soluble. The phylogenetic analysis clustered AChE1b together with other invertebrate AChEs belonging to type 1. However, AChE1b lacks some of the important amino acids for the function of a typical AChE. According to the docking analysis, *C. rogercresseyi* AChE1b could bind acetylcholine, azamethiphos and dichlorvos at different locations of the gorge. Enzymatic assays are necessary in order to properly identify the function of *C. rogercresseyi* AChE1b, but a scavenger function is possible, as is the case for vertebrate butyrylcholinesterase and for some AChE soluble forms occuring in invertebrates. In this respect, Kim et al. [58] described two AChEs in the honeybee, a membrane-anchored enzyme with a strong AChE activity and a soluble form with very low AChE activity. The authors showed that the inhibition of the main synaptic enzyme was reduced when inhibitors were pre-incubated with the soluble form. In the pinewood nematode, Kang et al. [59] also identified a soluble AChE form that conferred protection against anti-cholinesterase compounds.

Similar to *C. rogercresseyi*, two AChEs have been described in the other sea lice affecting farmed salmonids, *L. salmonis* [30]. These proteins were similar to each other and both grouped together with other invertebrate AChEs type 1. They were named type 1a and 1b. AChE1a was predicted to be the main synaptic AChE in *L. salmonis*. AChE1b has also the typical AChE signature and the important amino acids for the function of an AChE. It was predicted to be a soluble protein and was suggested to be a functionally active AChE.

As per the existing literature, decreased sensitivity towards organophosphates and carbamates is mostly associated with mutations in AChE genes in various arthropods (reviewed in [25, 28]). Among these, point mutations are the most commonly found. The majority of these missense mutations have been found in or around the active gorge of the enzyme, making it a hot spot for mutations. In order to correlate mutations or variants in AChE genes with organophosphate resistance, it is essential to properly characterize the sensitivity to the chemical of every single parasite used in the analysis. Bioassays designed for selecting individual lice are a reliable method to accomplish this [21, 22]. In our study, lice immobilized at 0.4 ppb or below were considered sensitive and lice unaffected at 2 ppb, resistant [7, 19, 22, 23]. At the louse population level, the bioassay performed in Farm 1 showed a louse sensitivity (EC_50_ ~0.5 ppb) close to the *C. rogercresseyi* putative naïve level (~0.15 ppb, see [7]). On the other hand, lice from Farm 2 showed reduced sensitivity towards azamethiphos (EC_50_ 1.2 ppb and no lice immobilized at 0.8 ppb or below). For reference, *L. salmonis* resistant strains have EC_50_ values between 2.1 and 3.7 ppb (24 h bioassay) [19, 21].

The main synaptic *C. rogercresseyi* AChE, AChE1a, was screened for variants in both azamethiphos-sensitive and -resistant lice in the present study. One non-synonymous change in the coding cDNA region, thymine to guanine at position 952, was found in the two *C. rogercresseyi* individuals surviving the highest azamethiphos concentration in the bioassay. This nucleotide change led to the amino acid change F318V (corresponding to F290 in *T. californica* AChE). F290 (*T. californica* AChE numbering) is a component part of the acyl pocket of AChE, which is responsible for the ligand specificity, and it is highly conserved in both AChE1 and AChE2 from various species, as shown in Fig. 2. F318 (*C. rogercresseyi* AChE1a numbering) is therefore an important residue, most likely playing an important role in the AChE function. Furthermore, F290 (*T. californica* AChE numbering) has been observed as one of the hot spots for mutations in AChE, conferring different levels of resistance towards organophosphates and carbamates in various arthropods (reviewed in [25, 26, 28]). The most common changes reported at that position are F to V, F to L (leucine) or F to Y (tyrosine). Several examples illustrate the association between the F290V change and organophosphate/carbamate resistant in arthropods. The mutation F416V (F290V, *T. californica* numbering) was found in organophosphate-resistant mosquitoes *Culex pipiens* from Cyprus [60]. Interestingly, when the authors introduced the F416V mutation in susceptible mosquitoes or in recombinant enzymes by site-directed mutagenesis, they found similar levels of resistance towards a range of organophosphates and carbamates when compared to the naturally mutated resistant mosquitoes [60, 61]. Resistant mosquitoes were homozygous for the mutation, and the activity of the mutated V416 AChE1 was similar to the activity of the susceptible wild enzyme (F416). The F416V mutation persisted for at least 16 years in the mosquito population, and it was especially protective against the organophosphate dichlorvos. Interestingly, salmonid fish infested with *C. rogercresseyi* had been treated with dichlorvos for 15 years in the past in the Chilean Los Lagos Region (1985–2000, reviewed in [8]). The dichlorvos exposure may well have selected the *C. rogercresseyi* AChE1a V318 variant in louse populations in the past, conferring some protection for azamethiphos in the present.

The same residue of the acyl binding pocket was also found mutated in other organophosphate and carbamate resistant arthropods. For example, the change F399V (homozygous) was reported in carbamate and organophosphate resistant codling moth, *Cydia pomonella* [62]. In carbamate resistant striped rice stemborers, *Chilo suppressalis*, the F402V variant was found alongside with four other mutations in AChE1 (both heterozygous and homozygous individuals were found) [63]. In the organophosphate resistant fall armyworm, *Spodoptera frugiperda*, Carvalho et al. [26] found three amino acid substitutions in the *ace-1* gene, one of them being F290V (*T. californica* numbering). Both heterozygous and homozygous individuals were found for this mutation. Overexpression of several genes was also found in organophosphate resistant fall armyworms. In the organophosphate resistant diamondback moth, *Plutella xylostella*, Yeh et al. [64] found three amino acid substitutions in AChE1. One of them was F386F/V (only heterozygous genotype was found). Their 3D modeling showed that the F386V change enlarges the space of the acetyl binding pocket. Our *C. rogercresseyi* AChE1a 3D model also revealed that the change from phenylalanine to valine at position 318 results in a wider active-site gorge.

The V318 variant in *C. rogercresseyi* AChE1a, has the same ten important amino acids as vertebrate BChE, the latter having valine or isoleucine (depending on the species, see Additional file 9) in position 290 (*T. californica* AChE numbering). Isoleucine and valine have similar biochemical properties (conservative variation). Vertebrate BChE has a wider substrate specificity than AChE, which could also be the case for the *C. rogercresseyi* AChE1a V318 variant. In invertebrates, Pezzementi et al. [65] investigated the acyl pocket of the amphioxus (*Branchiostoma floridae*) cholinesterase type 2 (ChE2) by site-directed mutagenesis. When the 312 phenylalanine of the acyl pocket (*B. floridae* numbering; F290 *T. californica* numbering) was replaced by isoleucine, a decrease in the substrate specificity of the enzyme was observed. In addition, the F312I mutation eliminated the substrate inhibition by ACh. The docking analysis performed in the present study showed that the F318V change in *C. rogercresseyi* AChE1a could modify the binding properties of azamethiphos to the enzyme. Enzymatic assays are though warranted in order to elucidate the activity of AChE1a V318 variant in the presence and absence of organophosphates.

Only heterozygous *C. rogercresseyi* individuals were found in our study. Several causes could explain the lack of homozygous lice. When the frequency of the variant in the population is low, the sample size must be large to detect homozygous individuals. The percentage of males carrying the F/V318 variant sampled before the azamethiphos field treatment in Farm 3 was very low (8.3%) and the percentaje of females was 50%, therefore the probability of finding homozygous individuals is low. The F318V change could also involve a high fitness cost for the homozygous variant. However, this change has been reported as homozygous in viable resistant individuals in other arthropods (reported earlier in the discussion). In our bioassays, one lice carrying the F/V318 variant was immobilized at a low azamethiphos concentration (Table 2). The heterozygous variant may not provide full protection against organophosphates, as shown by Kaur et al. [21, 22] in *L. salmonis*.

In order to investigate the association between the AChE1a F/V318 variant and azamethiphos sensitivity loss, 57 *C. rogercresseyi* collected before and after an azamethiphos field treatment were analysed. The analysis showed that every young adult female sampled from the fish three days after treatment carried the F/V318 variant (Table 3). These results indicate that the F/V318 variant plays an important role in protecting *C. rogercresseyi* against azamethiphos. However, other mechanisms could also be involved in the protection, since lice not carrying the F/V318 variant were unaffected at 1.2 ppb azamethiphos in the bioassays (Table 2). Behavioral avoidance, differences in the expression of the acetylcholinesterases and/or in the genes coding for detoxifying enzymes could contribute to the lice survival to azamethiphos exposure, as observed in other arthropods (reviewed in [24, 26–28]). Mobile small lice can hide under the scales of the fish, becoming less exposed to the bath treatment (C. Agusti and S. Bravo personal observation). In Farm 3, adult males without the F/V318 variant were found after the treatment. Mature adult males have higher mobility than adult females with fully developed genital segment. The post-treatment sampling occurred three days after the azamethiphos treatment, thus the mature adult males found post-treatment could be lice arriving from other untreated cages or farms.

In *L. salmonis*, azamethiphos is considered to have a poorer effect against the two chalimus stages compared to preadult and adult stages. In the present study with *C. rogercresseyi*, which has four chalimus stages, 57% efficacy was recorded for chalimus. This figure should be interpreted with caution as the first two chalimus stages are difficult to see and count correctly. Within the three days from treatment to sampling, some of the chalimus IV could have moulted to young adults. Still, if the survival of the treatment was independent of the F/V318 variant, an equal proportion of F318 and F/V318 would be expected in the surviving parasites, but instead 100% of the young females sampled after treatment had the F/V318 variant. Moreover, both young adult lice (one male and one female) unaffected at 2 ppb azamethiphos in the bioassay, were carrying the F/V318 variant.

*Caligus rogercresseyi* resistance towards azamethiphos is emerging and developing in Chile. In general, several resistance mechanisms can arise while resistance is developing. Although different mechanisms could be involved in the lice protection against azamethiphos, the AChE1a F/V318 variant is most probably playing a significant role in the observed reduced sensitivity. The variant could therefore be a good candidate as molecular marker, indicating loss of sensitivity in *C. rogercresseyi* towards organophosphates in Chile.

## Conclusions

The present study reports for the first time the identification and characterization of two AChEs in *C. rogercresseyi*. AChE1a is most likely the main synaptic AChE, whereas AChE1b could be a scavenger protein. We found one variant (F/V318) in *C. rogercresseyi* AChE1a that could play a role in making the parasite less sensitive to azamethiphos. This change has been reported to be conferring resistance in several other arthropods towards organophosphates and carbamates. An azamethiphos field treatment showed a selection of the F/V318 variant after treatment among young adult females, indicating an association between the presence of the variant and the survival of *C. rogercresseyi* to azamethiphos. Future screening in a larger sea lice population is warranted to validate this.

## Additional files

Additional file 1: Primers used for amplifying and sequencing the *Caligus rogercresseyi* AChE sequences found in this study. (PDF 16 kb)

Additional file 2: *Caligus rogercresseyi* AChE sequences in Whole-Genome Shotgun: contigs. (PDF 6 kb)

Additional file 3: Full list of abbreviations and UniprotKB database entry names from data used in Figs. 2 and 4 and Additional file 4. (PDF 21 kb)

Additional file 4: Phylogenetic analysis including *Caligus rogercresseyi* AChE1a and AChE1b together with several invertebrate and vertebrate carboxylesterases, esterases and other cholinesterases. Crustacean proteins are shown in red letters. Branches in red correspond to proteins tentatively assigned to the AChE type in the present analysis. For abbrevations and UniprotKB database entry names see Additional file 3. (PDF 170 kb)

Additional file 5: Alignment of the full coding *ace1a* cDNA sequence from 10 *Caligus rogercresseyi*. The lack of the star symbol under the sequences indicates a SNP. Every individual is represented by the two cDNA variants (a and b), according to the SNPs found. Individual louse code: resistant (LR1, LR2), reduced sensitivity (AL, BL, CL), sensitive (SS1, S1, S2, S3, S4). (PDF 118 kb)

Additional file 6: PDB file of the predicted 3D structure of *Caligus rogercresseyi* AChE1a F318 variant. Viewer: UCSF Chimera 1.10.2. software. https://www.cgl.ucsf.edu/chimera. (PDB 342 kb)

Additional file 7: PDB file of the predicted 3D structure of *Caligus rogercresseyi* AChE1a V318 variant. Viewer: UCSF Chimera 1.10.2. software. https://www.cgl.ucsf.edu/chimera. (PDB 331 kb)

Additional file 8: PDB file of the predicted 3D structure of *Caligus rogercresseyi* AChE1b. Viewer: UCSF Chimera 1.10.2. software. https://www.cgl.ucsf.edu/chimera. (PDB 296 kb)

Additional file 9: Alignment of six vertebrate butyrylcholinesterases (BChE), *Caligus rogercresseyi* AChE1a V318 variant (M) and *Caligus rogercresseyi* AChE1b. Figure showing a fragment of the alignment containing the amino acid corresponding to the position F290 (*Torpedo californica* AChE numbering) (highlighted). (PDF 165 kb)

## Abbreviations

3D: Three-dimensional
AChE: Acetylcholinesterase enzyme
ChE: Cholinesterase enzyme
EC_50_: The concentration of a chemical immobilizing 50% of the parasites
F318F/V: The important change found in the amino acid sequence in the main synaptic *Caligus rogercresseyi* AChE (AChE1a)
Ki: Concentration required to produce half-maximum inhibition (docking analysis)
MCMC: Markov Chain Monte Carlo analysis
MSA: Multiple Sequence Alignment
ORF: Open reading frame of a gene
ppb: μg l^-1^
QMEAN and GMQE: Global 3D model quality estimation scores
RMS: Root mean square value for carbon alpha (Cα) used for analyzing the fit between the selected templates and the predicted AChEs structures in the 3D analysis
UTR: Untranslated region of a gene
WGS: Whole-Genome Shotgun
