# Supplementary material for: Caligus rogercresseyi acetylcholinesterase types and variants: a potential marker for organophosphate resistance
Source: Parasit Vectors. 2018 Oct 30;11:570. doi: 10.1186/s13071-018-3151-7 (PMC6208076; doi:10.1186/s13071-018-3151-7)
Supplement: Supplementary file 1 — Primers used for amplifying and sequencing the Caligus rogercresseyi AChE sequences found in this study. (PDF 16 kb) [file 13071_2018_3151_MOESM1_ESM.pdf]

S1. Primers used for amplifying and sequencing the *Caligus rogercresseyi* AChE sequences found in this study.

Gene	Primer name	Primer sequence	Remarks
Cr_ace1a	WS2 Forward	CATCTTTCGTCGTGGGACTT	Amplifying the whole cDNA coding sequence (primer annealing temperature 62°C)
Cr_ace1a	WS9 Reverse	AGTGGGAGCGCCAATATAGA	Amplifying the whole cDNA coding sequence (primer annealing temperature 62°C)
Cr_ace1a	HS2 Forward	GGATTCTATTCCGGGACCTC	Internal primers for sequencing
Cr_ace1a	HS6 Forward	CAATTGTGGACGGGAGTCTT	Internal primers for sequencing
Cr_ace1a	WS7 Forward	CGAGTTCTTGAGGGTCTTCG	Internal primers for sequencing
Cr_ace1a	W25 Reverse	TGCAAAGGGGAAACAAACCG	Internal primers for sequencing
Cr_ace1a	W17 Reverse	TGCAGCATGACGGAGATGTT	Internal primers for sequencing
Cr_ace1a	HS1 Reverse	CGGGGATCGTATACTTCCAG	Internal primers for sequencing
Cr_ace1a	HS6 Reverse	CCCCACCTTGTTCACGTAGT	Internal primers for sequencing
Cr_ace1a	WS4 Reverse	GCCTCCGTAGATCCATACGA	Internal primers for sequencing
Cr_ace1a	HS4 Forward	GCATCTCCTTTCTCCCCTCT	Amplifying a fragment of the cDNA coding sequence for faster screening of the mutation
Cr_ace1a	HS6 Reverse	CCCCACCTTGTTCACGTAGT	Amplifying a fragment of the cDNA coding sequence for faster screening of the mutation
Cr_ace1b	T1 Forward	TGATGAGTTTGCGAGGACCA	Amplifying the whole cDNA coding sequence (primer annealing temperature 67°C)
Cr_ace1b	T1 Reverse	GTTGTTATGGGTGGGCACAG	Amplifying the whole cDNA coding sequence (primer annealing temperature 67°C)
Cr_ace1b	3 Forward	CAGTATCGATTGGGCAGCCT	Internal primers for sequencing
Cr_ace1b	3 Reverse	TGCCCATCATCTTCGTTCGT	Internal primers for sequencing
Cr_ace1b	4 Forward	ACGAACGAAGATGATGGGCA	Internal primers for sequencing
Cr_ace1b	6 Forward	AGTGCGTCTTCCAAGTGTCA	Internal primers for sequencing
Cr_ace1b	7 Forward	AGCACCTCATGACGTCACTA	Internal primers for sequencing
Cr_ace1b	8 Forward	CATTGGCAACCTTCGCTTCC	Internal primers for sequencing
Cr_ace1b	8 Reverse	AATTGGTCAAAGAGCCCCGC	Internal primers for sequencing
