# Supplementary material for: Caligus rogercresseyi acetylcholinesterase types and variants: a potential marker for organophosphate resistance
Source: Parasit Vectors. 2018 Oct 30;11:570. doi: 10.1186/s13071-018-3151-7 (PMC6208076; doi:10.1186/s13071-018-3151-7)
Supplement: Supplementary file 2 — Caligus rogercresseyi AChE sequences in Whole-Genome Shotgun: contigs. (PDF 6 kb) [file 13071_2018_3151_MOESM2_ESM.pdf]

S2. *C. rogercresseyi* AChE sequences in Whole-Genome Shotgun: contigs.

For GAZX01027370.1 cDNA sequence (ace1a):

gi|819763907|gb|LBBV01015450.1| Caligus rogercresseyi_Female_Contig_015470
gi|819393647|gb|LBBV01234453.1| Caligus rogercresseyi_ Female_Contig_234680
gi|819761156|gb|LBBV01017293.1| Caligus rogercresseyi_ Female_Contig_017313
gi|819536269|gb|LBBV01179936.1| Caligus rogercresseyi_ Female_Contig_180129
gi|819178740|gb|LBBU01052937.1| Caligus rogercresseyi_Male_Contig_052988
gi|819126630|gb|LBBU01105047.1| Caligus rogercresseyi Male_Contig_105143
gi|818985139|gb|LBBU01224471.1| Caligus rogercresseyi Male_Contig_224659

For GAZX01029466.1 cDNA sequence (ace1b):

gi|819685491|gb|LBBV01071544.1| Caligus rogercresseyi_Female_Contig_071634
gi|819685489|gb|LBBV01071545.1| Caligus rogercresseyi_Female_Contig_071635
gi|819771989|gb|LBBV01010092.1| Caligus rogercresseyi_Female_Contig_010110
gi|819126940|gb|LBBU01104737.1| Caligus rogercresseyi _Male_Contig_104833
gi|819013886|gb|LBBU01195724.1| Caligus rogercresseyi_Male_Contig_195887
gi|819114870|gb|LBBU01116807.1| Caligus rogercresseyi_Male_Contig_116909
