# Supplementary material for: Caligus rogercresseyi acetylcholinesterase types and variants: a potential marker for organophosphate resistance
Source: Parasit Vectors. 2018 Oct 30;11:570. doi: 10.1186/s13071-018-3151-7 (PMC6208076; doi:10.1186/s13071-018-3151-7)
Supplement: Supplementary file 3 — Full list of abbreviations and UniprotKB database entry names from data used in Figs. 2 and 4 and Additional file 4. (PDF 21 kb) [file 13071_2018_3151_MOESM3_ESM.pdf]

Additional file 3. Full list of abbreviations and UniprotKB database entry names from data used in Figs. 2 and 4 and Additional file 4.

__Insects:__ *Aedes albopictus* AO-AChE: Q2PGB1_AEDAL (A_albopictus_AChE2); *Aedes albopictus* AP-AChE: Q2PGB0_AEDAL (A_albopictus_AChE1); *Aedes albopictus* Putative acetylcholinesterase/butyrylcholinesterase: A0A023EWJ8|A0A023EWJ8_AEDAL (A_albopictus_ChE); *Anopheles gambiae* Ace: Q869C3|ACES_ANOGA (A_gambiae_AChE1); *Anopheles gambiae* AChE2: Q7QFG0_ANOGA (A_gambiae_AChE2); *Aphis gossypii* AChE1: Q65YU0_APHGO (A_gossypii_AChE2); *Aphis gossypii* Ace2: Q65Z60_APHGO (A_gossypii_AChE1); *Apis mellifera* Ace 1: A0A087ZWF0_APIME (A_mellifera_AChE1); *Apis mellifera* Ace 2: Q4LEZ2_APIME (A_mellifera_AChE2); *Apis mellifera* Venom carboxylesterase 6: B2D0J5|EST6_APIME (A_mellifera_VCarEst6); *Anopheles stephensi* AChE: P56161|ACES_ANOST (A_stephensi_AChE2); *Bactrocera oleae* Carboxylic ester hydrolase: Q8MVZ4|Q8MVZ4_BACOL (B_oleae_CarEstHy); *Bemisia tabaci* ace1: B3SST3_BEMTA (B_tabaci_AChE1); *Bemisia tabaci* ace2: B3SST5_BEMTA (B_tabaci_AChE2); *Blattella germánica* Ace 1: Q2PZG3_BLAGE (B_germanica_AChE1); *Blattella germanica* Ace 2: Q2PZB3_BLAGE (B_germanica_AChE2); *Bombyx mandarina* ace1: A2IBS5_BOMMA (B_mandarina_AChE1); *Bombyx mandarina* ace: A1Z267_BOMMA (B_mandarina_AChE2); *Bombyx mori* ace1: B0FGT1_BOMMO (B_mori_AChE1); *Bombyx mori* ace2: B0FGT2_BOMMO (B_mori_AChE2); *Bombyx mori* Carboxylic ester hydrolase: E1CGA5|E1CGA5_BOMMO (B_mori_CarEstHy5); *Bombyx mori* Carboxylic ester hydrolase: E1CGA6|E1CGA6_BOMMO (B_mori_CarEstHy6); *Ctenocephalides felis* ace1: D2U6X5_CTEFE (C_felis_AChE1); *Ctenocephalides felis* ace2: D2U6X6_CTEFE (C_felis_AChE2); *Cimex lectularius* Ace 1: G3LUX3_CIMLE (C_lectularius_AChE1); *Cimex lectularius* Ace 2: G1K820_CIMLE (C_lectularius_AChE2); *Cimex lectularius* Carboxylic ester hydrolase: G1K818|G1K818_CIMLE (C_lectularius_CarEstHy); *Culex quinquefasciatus* Cholinesterase: B0WDB9|B0WDB9_CULQU (C_quinquefasciatus_ChE); *Culex pipiens* ACHE1: Q86GC8|ACES_CULPI (C_pipiens_AChE1); *Culex pipiens* ace-2: Q1JS07_CULPI (C_pipiens_AChE2); *Chilo suppressalis* ace1: A5HBN8_CHISP (C_suppressalis_AChE1); *Chilo suppressalis* AChE2: B2BD82_CHISP (C_suppressalis_AChE2); *Drosophila mauritiana* Esterase 6: P47982|EST6_DROMA (D_mauritiana _Est6); *Drosophila melanogaster* AChE: P07140|ACES_DROME (D_melanogaster_AChE); *Drosophila melanogaster* Esterase 6: P08171|EST6_DROME (D_melanogaster_Est6); *Drosophila melanogaster* Esterase P: P18167|ESTP_DROME (D_melanogaster_EstP); *Drosophila melanogaster* Palmitoleoyl-protein carboxylesterase NOTUM: Q9VUX3|NOTUM_DROME (D_melanogaster_PalmCar_N); *Drosophila miranda* Esterase 5B: O16170|EST5B_DROMI (D_miranda_Est5B); *Drosophila miranda* Esterase 5A: O16168|EST5A_DROMI (D_miranda_Est5A); *Drosophila miranda* Esterase 5C: O16169|EST5C_DROMI (D_miranda_Est5C); *Drosophila persimilis* Esterase 5A: O16173|EST5A_DROPE (D_persimilis_Est5A); *Drosophila persimilis* Esterase 5C: O16171|EST5C_DROPE (D_persimilis_Est5C); *Drosophila persimilis* Esterase 5B:

O16172|EST5B_DROPE (D_persimilis_Est5B); *Drosophila pseudoobscura pseudoobscura* Esterase 5A: P25727|EST5A_DROPS (D_psob_psob_Est5A); *Drosophila pseudoobscura pseudoobscura* Esterase 5B: P25726|EST5B_DROPS (D_psob_psob_Est5B); *Drosophila pseudoobscura pseudoobscura* Esterase 5C: P25725|EST5C_DROPS (D_psob_psob_Est5C); *Drosophila simulans* Esterase 6: Q08662|EST6_DROSI (D_simulans_Est6); *Drosophila virilis* Esterase S: Q05487|ESTS_DROVI (D_virilis_EstS); *Leptinotarsa decemlineata* ace1: F8TTP2_LEPDE (L_decemlineata_AChE1); *Leptinotarsa decemlineata* AChE: Q27677|ACES_LEPDE (L_decemlineata_AChE2); *Liposcelis entomophila* Ace_1: B6D7G8_9NEOP (L_entomophila_AChE1); *Liposcelis entomophila* Ace_2: B6D7G9_9NEOP (L_entomophila_AChE2); *Musca domestica* AChE: Q8MXC4_MUSDO (M_domestica_AChE); *Musca domestica* Carboxylic ester hydrolase: T1PHU7|T1PHU7_MUSDO (M_domestica_CarEstHy); *Nephotettix cincticeps* AO-AChE: Q9NJH6_NEPCI (N_cincticeps_AChE2); *Nephotettix cincticeps* AP-AChE: D2KTI9_NEPCI (N_cincticeps_AChE1).

Crustaceans: *Lepeophtheirus salmonis* ace1a: A0A0A7E9D3_LEPSM (L_salmonis_AChE1a); *Lepeophtheirus salmonis* ace1b: A0A0A7E972_LEPSM (L_salmonis_AChE1b); *Lepeophtheirus salmonis* putative carboxylic ester hydrolase 1: A0A0K2SXR6|A0A0K2SXR6_LEPSM (L_salmonis_CarEstHy1); *Lepeophtheirus salmonis* put2carboxy, uncharacterized protein: A0A0K2TUC8|A0A0K2TUC8_LEPSM (L_salmonis_Car2); *Lepeophtheirus salmonis* put3carboxy CG4382 CG4382PAlike [Tribolium castaneum] Carboxylesterase B: A0A0K2T105|A0A0K2T105_LEPSM (L_salmonis_Car3); *Lepeophtheirus salmonis* Venom carboxylesterase 6 like [Bombus impatiens]: A0A0K2VCP5|A0A0K2VCP5_LEPSM (L_salmonis_VCarEst6_Bi); *Tigriopus japonicus* AChE1: A0A0A0QUZ0_TIGJA (T_japonicus_AChE1); *Tigriopus japonicus* AChE2: A0A0A0QWL0_TIGJA (T_japonicus_AChE2).

Nematodes: *Bursaphelenchus xylophilus* ace-1: D2JYW9_BURXY (B_xylophilus_AChE1); *Bursaphelenchus xylophilus* ace-2: C5IAX1_BURXY (B_xylophilus_AChE2); *Bursaphelenchus xylophilus* ace-3: D2JYX1_BURXY (B_xylophilus_AChE3); *Caenorhabditis briggsae* ace-1: Q27459|ACE1_CAEBR (C_briggsae_AChE1); *Caenorhabditis briggsae* ace-2: O61378_CAEBR (C_briggsae_AChE2); *Caenorhabditis briggsae* ace-3: Q9NDG9_CAEBR (C_briggsae_AChE3); *Caenorhabditis briggsae* ace-4: Q9NDG8|ACE4_CAEBR (C_briggsae_AChE4); *Caenorhabditis briggsae* Gut esterase 1: Q04456|EST1_CAEBR (C_briggsae_Est1); *Caenorhabditis elegans* ace-1: P38433|ACE1_CAEEL (C_elegans_AChE1); *Caenorhabditis elegans* ace-2: G5EDV9_CAEEL (C_elegans_AChE2); *Caenorhabditis elegans* ace-3: Q9U295_CAEEL (C_elegans_AChE3); *Caenorhabditis elegans* ace-4: G5EDN1_CAEEL (C_elegans_AChE4); *Caenorhabditis elegans* Esterase CM06B1: Q07085|EST2_CAEEL (C_elegans_Est2); *Caenorhabditis elegans* Gut esterase 1: Q04457|EST1_CAEEL (C_elegans_Est1).

Arachnida (ticks): *Amblyomma triste* Putative acetylcholinesterase/butyrylcholinesterase: A0A023GPC3|A0A023GPC3_9ACAR (A_triste_ChE); *Ixodes ricinus* Putative acetylcholinesterase/butyrylcholinesterase 1: V5H4P2|V5H4P2_IXORI (I_ricinus_ChE1); *Ixodes ricinus* Putative acetylcholinesterase/butyrylcholinesterase 2: A0A0K8R3H1|A0A0K8R3H1_IXORI (I_ricinus_ChE2); *Ixodes ricinus* Putative acetylcholinesterase/butyrylcholinesterase 3: A0A0K8RAC7|A0A0K8RAC7_IXORI (I_ricinus_ChE3); *Ixodes ricinus* Putative acetylcholinesterase/butyrylcholinesterase 4: A0A0K8RMR3|A0A0K8RMR3_IXORI (I_ricinus_ChE4); *Ixodes scapularis* Acetylcholinesterase/butyrylcholinesterase, putative: B7PU15|B7PU15_IXOSC (I_scapularis_ChE); *Rhipicephalus decoloratus* AChE: O61987_RHIDE (R_decoloratus_AChE); *Rhipicephalus microplus* ace1: O45210_RHIMP (R_microplus_AChE); *Rhipicephalus microplus* Carboxylic ester hydrolase AChE3: E0YCF7|E0YCF7_RHIMP (R_microplus_AChE3); *Rhipicephalus microplus* Carboxylic ester hydrolase Ace2put: O61864|O61864_RHIMP (R_microplus_Ace2); *Rhipicephalus pulchellus* Putative acetylcholinesterase/butyrylcholinesterase 1: L7M115|L7M115_9ACAR (R_pulchellus_ChE1); *Rhipicephalus pulchellus* Putative acetylcholinesterase/butyrylcholinesterase 2: L7M799|L7M799_9ACAR (R_pulchellus_ChE2);

Arachnida (others): *Tetranychus urticae* (mite) AchE1: D8V7K2_TETUR (T_urticae_AChE); *Tetranychus urticae* Carboxylic ester hydrolase 1: T1KX09|T1KX09_TETUR (T_urticae_CarEstHy1); *Tetranychus urticae* Carboxylic ester hydrolase 2: T1L404|T1L404_TETUR (T_urticae_CarEstHy2); *Trittame loki* (spider) AchE1: W4VSJ0|ACES_TRILK (T_loki_AChE).

Trematode: *Schistosoma haematobium* AChE: Q86GL8_SCHHA (S_haematobium_AChE); *Schistosoma japonicum* Butyrylcholinesterase: C1L3S3|C1L3S3_SCHJA (S_japonicum_BChE).

Vertebrates: *Bungarus fasciatus* AChE: Q92035|ACES_BUNFA (B_fasciatus_AChE); *Bos taurus* AChE: P23795|ACES_BOVIN (B_taurus_AChE); *Bos taurus* BChE: P32749|CHLE_BOVIN (B_taurus_BChE); *Bos taurus* Carboxylesterase 4A: P0C6R3|EST4A_BOVIN (B_taurus_CarEst_4A); *Canis familiaris* Carboxylesterase 5A: Q6AW47|EST5A_CANFA (C_familiaris_CarEst_5A); *Danio rerio* AChE: Q9DDE3|ACES_DANRE (D_rerio_AChE); *Danio rerio* Carboxylesterase notum2: E7F0Z8|NOTU2_DANRE (D_rerio_CarEst_N2); *Electrophorus electricus* AChE: O42275|ACES_ELEEL (E_electricus_AChE); *Felis catus* AChE: O62763|ACES_FELCA (F_catus_AChE); *Felis catus* BChE: O62760|CHLE_FELCA (F_catus_BChE); *Felis catus* Carboxylesterase 5A: Q8I034|EST5A_FELCA (F_catus_CarEst_5A); *Gallus gallus* BChE: Q90ZK8|Q90ZK8_CHICK (G_gallus_BChE); *Homo sapiens* AChE: P22303|ACES_HUMAN (H_sapiens_AChE); *Homo sapiens* BChE: P06276|CHLE_HUMAN (H_sapiens_BChE); *Homo sapiens* Carboxylesterase 3: Q6UWW8|EST3_HUMAN (H_sapiens_CarEst_3); *Homo sapiens* Carboxylesterase 5A: Q6NT32|EST5A_HUMAN (H_sapiens_CarEst_5A); *Homo sapiens* Liver carboxylesterase 1: P23141|EST1_HUMAN

(H_sapiens_LiCarEst_1); *Homo sapiens* Cocaine_esterase: O00748|EST2_HUMAN (H_sapiens_CoEst); *Mus musculus* AChE: P21836|ACES_MOUSE (M_musculus_AChE); *Mus musculus* BChE: Q03311|CHLE_MOUSE (M_musculus_BChE); *Mus musculus* Carboxylesterase_1D: Q8VCT4|CES1D_MOUSE (M_musculus_CarEst_1D); *Mus musculus* Carboxylesterase_1C: P23953|EST1C_MOUSE (M_musculus_CarEst_1C); *Mus musculus* Carboxylesterase_1E: Q64176|EST1E_MOUSE (M_musculus_CarEst_1E); *Mus musculus* Liver carboxylesterase 1: Q8VCC2|EST1_MOUSE (M_musculus_LiCarEst_1); *Mus musculus* Carboxylesterase 3A: Q63880|EST3A_MOUSE (M_musculus_CarEst_3A); *Mus musculus* Carboxylesterase_3B: Q8VCU1|EST3B_MOUSE (M_musculus_CarEst_3B); *Mus musculus* Carboxylesterase_5A: Q6AW46|EST5A_MOUSE (M_musculus_CarEst_5A); *Oryctolagus cuniculus* BChE: P21927|CHLE_RABIT (O_cuniculus_BChE); *Rattus norvegicus* Carboxylesterase 1D: P16303|CES1D_RAT (R_norvegicus_CarEst_1D); *Rattus norvegicus* Carboxylesterase 1C: P10959|EST1C_RAT (R_norvegicus_CarEst_1C); *Rattus norvegicus* Carboxylesterase 1E: Q63108|EST1E_RAT (R_norvegicus_CarEst_1E); *Rattus norvegicus* Liver carboxylesterase 4: Q64573|EST4_RAT (R_norvegicus_LiCarEst_4); *Rattus norvegicus* Carboxylesterase 5A: Q5GRG2|EST5A_RAT (R_norvegicus_CarEst_5A); *Rattus norvegicus* Liver carboxylesterase B-1: Q63010|EST5_RAT (R_norvegicus_LiCarEst_B1); *Torpedo californica* AChE: P04058|ACES_TORCA (T_californica_AChE).
