# Supplementary figures and images for: Caligus rogercresseyi acetylcholinesterase types and variants: a potential marker for organophosphate resistance
Source: Parasit Vectors. 2018 Oct 30;11:570. doi: 10.1186/s13071-018-3151-7 (PMC6208076; doi:10.1186/s13071-018-3151-7)

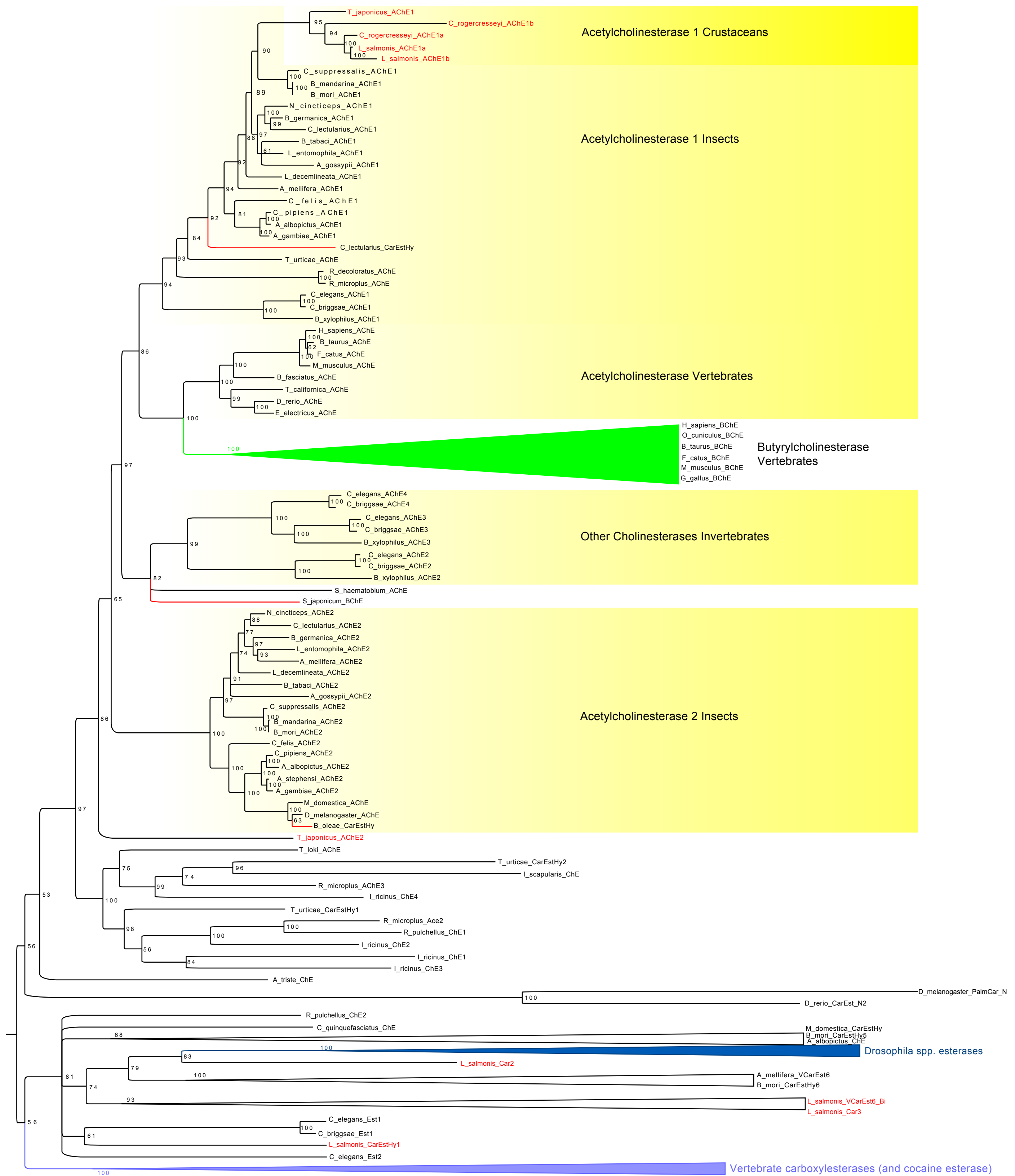

Supplement: Supplementary file 4 — Phylogenetic analysis including Caligus rogercresseyi AChE1a and AChE1b together with several invertebrate and vertebrate carboxylesterases, esterases and other cholinesterases. Crustacean proteins are shown in red letters. Branches in red correspond to proteins tentatively assigned to the AChE type in the present analysis. For abbrevations and UniprotKB database entry names see Additional file 3. (PDF 170 kb) [file 13071_2018_3151_MOESM4_ESM.pdf]
