# Supplementary material for: Caligus rogercresseyi acetylcholinesterase types and variants: a potential marker for organophosphate resistance
Source: Parasit Vectors. 2018 Oct 30;11:570. doi: 10.1186/s13071-018-3151-7 (PMC6208076; doi:10.1186/s13071-018-3151-7)
Supplement: Supplementary file 5 — Alignment of the full coding ace1a cDNA sequence from 10 Caligus rogercresseyi. The lack of the star symbol under the sequences indicates a SNP. Every individual is represented by the two cDNA variants (a and b), according to the SNPs found. Individual louse code: resistant (LR1, LR2), reduced sensitivity (AL, BL, CL), sensitive (SS1, S1, S2, S3, S4). (PDF 118 kb) [file 13071_2018_3151_MOESM5_ESM.pdf]

Additional file 5. Alignment of the full coding acetylcholinesterase ace1a cDNA sequence from 10 *Caligus rogercresseyi* individuals using CLUSTAL O (1.2.2) multiple sequence alignment. Several SNPs were found, but only two led to amino acid changes (T952G, bold and underlined, and G82A). The lack of the star symbol under the sequences indicates a SNP. Every individual is represented by the two cDNA variants (a and b), according with the SNPs found. Individual louse code: Resistant (LR1, LR2), reduced sensitivity (AL, BL, CL), sensitive (SS1, S1, S2, S3, S4).

```
LR2_b      atgcatattcaaaaagtcagaggggcttacttcgataagctattagtgtatttgctcact
CL_b       atgcatattcaaaaagtcagaggggcttacttcgataagctattagtgtatttgctcact
S3_b       atgcatattcaaaaagtcagaggggcttacttcgataagctattagtgtatttgctcact
LR1_b      atgcatattcaaaaagtcagaggggcttacttcgataagctattagtgtatttgctcact
LR2_a      atgcatattcaaaaagtcagaggggcttacttcgataagctattagtgtatttgctcact
S2_a       atgcatattcaaaaagtcagaggggcttacttcgataagctattagtgtatttgctcact
S4_b       atgcatattcaaaaagtcagaggggcttacttcgataagctattagtgtatttgctcact
SS1_a      atgcatattcaaaaagtcagaggggcttacttcgataagctattagtgtatttgctcact
S4_a       atgcatattcaaaaagtcagaggggcttacttcgataagctattagtgtatttgctcact
S2_b       atgcatattcaaaaagtcagaggggcttacttcgataagctattagtgtatttgctcact
BL_b       atgcatattcaaaaagtcagaggggcttacttcgataagctattagtgtatttgctcact
BL_a       atgcatattcaaaaagtcagaggggcttacttcgataagctattagtgtatttgctcact
AL_a       atgcatattcaaaaagtcagaggggcttacttcgataagctattagtgtatttgctcact
S3_a       atgcatattcaaaaagtcagaggggcttacttcgataagctattagtgtatttgctcact
S1_b       atgcatattcaaaaagtcagaggggcttacttcgataagctattagtgtatttgctcact
CL_a       atgcatattcaaaaagtcagaggggcttacttcgataagctattagtgtatttgctcact
AL_b       atgcatattcaaaaagtcagaggggcttacttcgataagctattagtgtatttgctcact
LR1_a      atgcatattcaaaaagtcagaggggcttacttcgataagctattagtgtatttgctcact
S1_a       atgcatattcaaaaagtcagaggggcttacttcgataagctattagtgtatttgctcact
SS1_b      atgcatattcaaaaagtcagaggggcttacttcgataagctattagtgtatttgctcact
           ************************************************************

LR2_b      ttgtcatggagcgtgggtgctgtcgttcaggatgacctggtgattacgacgaagaaagga
CL_b       ttgtcatggagcgtgggtgctgtcgttcaggatgacctggtgattacgacgaagaaagga
S3_b       ttgtcatggagcgtgggtgctgtcgttcaggatgacctggtgattacgacgaagaaagga
LR1_b      ttgtcatggagcgtgggtgctgtcgttcaggatgacctggtgattacgacgaagaaagga
LR2_a      ttgtcatggagcgtgggtgctgtcgttcaggatgacctggtgattacgacgaagaaagga
S2_a       ttgtcatggagcgtgggtgctgtcgttcaggatgacctggtgattacgacgaagaaagga
S4_b       ttgtcatggagcgtgggtgctgtcgttcaggatgacctggtgattacgacgaagaaagga
SS1_a      ttgtcatggagcgtgggtgctatcgttcaggatgacctggtgattacgacgaagaaagga
S4_a       ttgtcatggagcgtgggtgctgtcgttcaggatgacctggtgattacgacgaagaaagga
S2_b       ttgtcatggagcgtgggtgctgtcgttcaggatgacctggtgattacgacgaagaaagga
BL_b       ttgtcatggagcgtgggtgctgtcgttcaggatgacctggtgattacgacgaagaaagga
BL_a       ttgtcatggagcgtgggtgctgtcgttcaggatgacctggtgattacgacgaagaaagga
AL_a       ttgtcatggagcgtgggtgctgtcgttcaggatgacctggtgattacgacgaagaaagga
S3_a       ttgtcatggagcgtgggtgctgtcgttcaggatgacctggtgattacgacgaagaaagga
S1_b       ttgtcatggagcgtgggtgctgtcgttcaggatgacctggtgattacgacgaagaaagga
CL_a       ttgtcatggagcgtgggtgctgtcgttcaggatgacctggtgattacgacgaagaaagga
AL_b       ttgtcatggagcgtgggtgctgtcgttcaggatgacctggtgattacgacgaagaaagga
LR1_a      ttgtcatggagcgtgggtgctgtcgttcaggatgacctggtgattacgacgaagaaagga
S1_a       ttgtcatggagcgtgggtgctgtcgttcaggatgacctggtgattacgacgaagaaagga
SS1_b      ttgtcatggagcgtgggtgctgtcgttcaggatgacctggtgattacgacgaagaaagga
           ********************* **************************************
```

```
LR2_b       aagatccgaggagtcacgctcaaatccgctacaaatagggaagtggatgcatggtatggt
CL_b        aagatccgaggagtcacgctcaaatccgctacaaatagggaagtggatgcatggtatggt
S3_b        aagatccgaggagtcacgctcaaatccgctacaaatagggaagtggatgcatggtatggt
LR1_b       aagatccgaggagtcacgctcaaatccgctacaaatagggaagtggatgcatggtatggt
LR2_a       aagatccgaggagtcacgctcaaatccgctacaaatagggaagtggatgcatggtatggt
S2_a        aagatccgaggagtcacgctcaaatccgctacaaatagggaagtggatgcatggtatggt
S4_b        aagatccgaggagtcacgctcaaatccgctacaaatagggaagtggatgcatggtatggt
SS1_a       aagatccgaggagtcacgctcaaatccgctacaaatagggaagtggatgcatggtatggt
S4_a        aagatccgaggagtcacgctcaaatccgctacaaatagggaagtggatgcatggtatggt
S2_b        aagatccgaggagtcacgctcaaatccgctacaaatagggaagtggatgcatggtatggt
BL_b        aagatccgaggagtcacgctcaaatccgctacaaatagggaagtggatgcatggtatggt
BL_a        aagatccgaggagtcacgctcaaatccgctacaaatagggaagtggatgcatggtatggt
AL_a        aagatccgaggagtcacgctcaaatccgctacaaatagggaagtggatgcatggtatggt
S3_a        aagatccgaggagtcacgctcaaatccgctacaaatagggaagtggatgcatggtatggt
S1_b        aagatccgaggagtcacgctcaaatccgctacaaatagggaagtggatgcatggtatggt
CL_a        aagatccgaggagtcacgctcaaatccgctacaaatagggaagtggatgcatggtatggt
AL_b        aagatccgaggagtcacgctcaaatccgctacaaatagggaagtggatgcatggtatggt
LR1_a       aagatccgaggagtcacgctcaaatccgctacaaatagggaagtggatgcatggtatggt
S1_a        aagatccgaggagtcacgctcaaatccgctacaaatagggaagtggatgcatggtatggt
SS1_b       aagatccgaggagtcacgctcaaatccgctacaaatagggaagtggatgcatggtatggt
            ************************************************************

LR2_b       attccttatgctcagcccccctgtgggcaaccttcgcttccgtcacccgcgccacattgac
CL_b        attccttatgctcagcccccctgtgggcaaccttcgcttccgtcacccgcgccacattgac
S3_b        attccttatgctcagcccccctgtgggcaaccttcgcttccgtcacccgcgccacattgac
LR1_b       attccttatgctcagcccccctgtgggcaaccttcgcttccgtcacccgcgccacattgac
LR2_a       attccttatgctcagcccccctgtgggcaaccttcgcttccgtcacccgcgccacattgac
S2_a        attccttatgctcagcccccctgtgggcaaccttcgcttccgtcacccgcgccacattgac
S4_b        attccttatgctcagcccccctgtgggcaaccttcgcttccgtcacccgcgccacattgac
SS1_a       attccttatgctcagcccccctgtgggcaaccttcgcttccgtcacccgcgccacattgac
S4_a        attccttatgctcagcccccctgtgggcaaccttcgcttccgtcacccgcgccacattgac
S2_b        attccttatgctcagcccccctgtgggcaaccttcgcttccgtcacccgcgccacattgac
BL_b        attccttatgctcagcccccctgtgggcaaccttcgcttccgtcacccgcgccacattgac
BL_a        attccttatgctcagcccccctgtgggcaaccttcgcttccgtcacccgcgccacattgac
AL_a        attccttatgctcagcccccctgtgggcaaccttcgcttccgtcacccgcgccacattgac
S3_a        attccttatgctcagcccccctgtgggcaaccttcgcttccgtcacccgcgccacattgac
S1_b        attccttatgctcagcccccctgtgggcaaccttcgcttccgtcacccgcgccacattgac
CL_a        attccttatgctcagcccccctgtgggcaaccttcgcttccgtcacccgcgccacattgac
AL_b        attccttatgctcagcccccctgtgggcaaccttcgcttccgtcacccgcgccacattgac
LR1_a       attccttatgctcagcccccctgtgggcaaccttcgcttccgtcacccgcgccacattgac
S1_a        attccttatgctcagcccccctgtgggcaaccttcgcttccgtcacccgcgccacattgac
SS1_b       attccttatgctcagcccccctgtgggcaaccttcgcttccgtcacccgcgccacattgac
            ************************************************************

LR2_b       gcctgggaaggcatcaaggagacgacgcgacccccaaactcttgcgtccaagtcgtggac
CL_b        gcctgggaaggcatcaaggagacgacgcgacccccaaactcttgcgtccaagtcgtggac
S3_b        gcctgggaaggcatcaaggagacgacgcgacccccaaactcttgcgtccaagtcgtggac
LR1_b       gcctgggaaggcatcaaggagacgacgcgacccccaaactcttgcgtccaagtcgtggac
LR2_a       gcctgggaaggcatcaaggagacgacgcgacccccaaactcttgcgtccaagtcgtggac
S2_a        gcctgggaaggcatcaaggagacgacgcgacccccaaactcttgcgtccaagtcgtggac
S4_b        gcctgggaaggcatcaaggagacgacgcgacccccaaactcttgcgtccaagtcgtggac
SS1_a       gcctgggaaggcatcaaggagacgacgcgacccccaaactcttgcgtccaagtcgtggac
S4_a        gcctgggaaggcatcaaggagacgacgcgacccccaaactcttgcgtccaagtcgtggac
S2_b        gcctgggaaggcatcaaggagacgacgcgacccccaaactcttgcgtccaagtcgtggac
BL_b        gcctgggaaggcatcaaggagacgacgcgacccccaaactcttgcgtccaagtcgtggac
BL_a        gcctgggaaggcatcaaggagacgacgcgacccccaaactcttgcgtccaagtcgtggac
AL_a        gcctgggaaggcatcaaggagacgacgcgacccccaaactcttgcgtccaagtcgtggac
S3_a        gcctgggaaggcatcaaggagacgacgcgacccccaaactcttgcgtccaagtcgtggac
S1_b        gcctgggaaggcatcaaggagacgacgcgacccccaaactcttgcgtccaagtcgtggac
CL_a        gcctgggaaggcatcaaggagacgacgcgacccccaaactcttgcgtccaagtcgtggac
AL_b        gcctgggaaggcatcaaggagacgacgcgacccccaaactcttgcgtccaagtcgtggac
LR1_a       gcctgggaaggcatcaaggagacgacgcgacccccaaactcttgcgtccaagtcgtggac
S1_a        gcctgggaaggcatcaaggagacgacgcgacccccaaactcttgcgtccaagtcgtggac
SS1_b       gcctgggaaggcatcaaggagacgacgcgacccccaaactcttgcgtccaagtcgtggac
            ************************************************************
```

```
LR2_b       acgctcttccccggtttcgaaggggcagaaatgtggaacatcaacacggagccaagcgag
CL_b        acgctcttccccggtttcgaaggggcagaaatgtggaacatcaacacggagccaagcgag
S3_b        acgctcttccccggtttcgaaggggcagaaatgtggaacatcaacacggagccaagcgag
LR1_b       acgctcttccccggtttcgaaggggcagaaatgtggaacatcaacacggagccaagcgag
LR2_a       acgctcttccccggtttcgaaggggcagaaatgtggaacatcaacacggagccaagcgag
S2_a        acgctcttccccggtttcgaaggggcagaaatgtggaacatcaacacggagccaagcgag
S4_b        acgctcttccccggtttcgaaggggcagaaatgtggaacatcaacacggagccaagcgag
SS1_a       acgctcttccccggtttcgaaggggcagaaatgtggaacatcaacacggagccaagcgag
S4_a        acgctttttccccggtttcgaaggggcagaaatgtggaacatcaacacggagccaagcgag
S2_b        acgctcttccccggtttcgaaggggcagaaatgtggaacatcaacacggagccaagcgag
BL_b        acgctcttccccggtttcgaaggggcagaaatgtggaacatcaacacggagccaagcgag
BL_a        acgctcttccccggtttcgaaggggcagaaatgtggaacatcaacacggagccaagcgag
AL_a        acgctcttccccggtttcgaaggggcagaaatgtggaacatcaacacggagccaagcgag
S3_a        acgctcttccccggtttcgaaggggcagaaatgtggaacatcaacacggagccaagcgag
S1_b        acgctcttccccggtttcgaaggggcagaaatgtggaacatcaacacggagccaagcgag
CL_a        acgctcttccccggtttcgaaggggcagaaatgtggaacatcaacacggagccaagcgag
AL_b        acgctcttccccggtttcgaaggggcagaaatgtggaacatcaacacggagccaagcgag
LR1_a       acgctcttccccggtttcgaaggggcagaaatgtggaacatcaacacggagccaagcgag
S1_a        acgctcttccccggtttcgaaggggcagaaatgtggaacatcaacacggagccaagcgag
SS1_b       acgctcttccccggtttcgaaggggcagaaatgtggaacatcaacacggagccaagcgag
            ***** ******************************************************

LR2_b       gactgcctctacctaagcgtccacgtccccaagcctagacccacaggctccgccgtcctc
CL_b        gactgcctctacctaagcgtccacgtccccaagcctagacccacaggctccgccgtcctc
S3_b        gactgcctctacctaagcgtccacgtccccaagcctagacccacaggctccgccgtcctc
LR1_b       gactgcctctacttaagcgtgcacgtccccaagcctagacccacaggctccgccgtcctc
LR2_a       gactgcctctacttaagcgtgcacgtccccaagcctagacccacaggctccgccgtcctc
S2_a        gactgcctctacttaagcgtccacgtccccaagcctagacccacaggctccgccgtcctc
S4_b        gactgcctctacctaagcgtccacgtccccaagcctagacccacaggctccgccgtcctc
SS1_a       gactgcctctacctaagcgtccacgtccccaagcctagacccacaggctccgccgtcctc
S4_a        gactgcctctacctaagcgtccacgtccccaagcctagacccacaggctccgccgtcctc
S2_b        gactgcctctacctaagcgtgcacgtccccaagcctagacccacaggctccgccgtcctc
BL_b        gactgcctctacctaagcgtccacgtccccaagcctagacccacaggctccgccgtcctc
BL_a        gactgcctctacctaagcgtccacgtccccaagcctagacccacaggctccgccgtcctc
AL_a        gactgcctctacctaagcgtccacgtccccaagcctagacccacaggctccgccgtcctc
S3_a        gactgcctctacctaagcgtccacgtccccaagcctagacccacaggctccgccgtcctc
S1_b        gactgcctctacctaagcgtccacgtccccaagcctagacccacaggctccgccgtcctc
CL_a        gactgcctctacctaagcgtccacgtccccaagcctagacccacaggctccgccgtcctg
AL_b        gactgcctctacctaagcgtccacgtccccaagcctagacccacaggctccgccgtcctc
LR1_a       gactgcctctacctaagcgtccacgtccccaagcctagacccacaggctccgccgtcctc
S1_a        gactgcctctacctaagcgtccacgtccccaagcctagacccacaggctccgccgtcctc
SS1_b       gactgcctctacctaagcgtccacgtccccaagcctagacccacaggctccgccgtcctc
            ************ ******* **************************************

LR2_b       gtatggatctacggaggcggattctattccgggacctccaccctggaagtatacgatccc
CL_b        gtatggatctacggaggcggattctattccgggacctccaccctggaagtatacgatccc
S3_b        gtatggatctacggaggcggattctattccgggacctccaccctggaagtatacgatccc
LR1_b       gtatggatctacggaggcggattctattccgggacctccaccctggaagtatacgatccc
LR2_a       gtatggatctacggaggcggattctattccgggacctccaccctggaagtatacgatccc
S2_a        gtatggatctacggaggcggattctattccgggacctccaccctggaagtatacgatccc
S4_b        gtatggatctacggaggcggattctattccgggacctccaccctggaagtatacgatccc
SS1_a       gtatggatctacggaggcggattctattccgggacctccaccctggaagtatacgatccc
S4_a        gtatggatctacggaggcggattctattccgggacctccaccctggaagtatacgatccc
S2_b        gtatggatctacggaggcggattctattccgggacctccaccctggaagtatacgatccc
BL_b        gtatggatctacggaggcggattctattccgggacctccaccctggaagtatacgatccc
BL_a        gtatggatctacggaggcggattctattccgggacctccaccctggaagtatacgatccc
AL_a        gtatggatctacggaggcggattctattccgggacctccaccctggaagtatacgatccc
S3_a        gtatggatctacggaggcggattctattccgggacctccaccctggaagtatacgatccc
S1_b        gtatggatctacggaggcggattctattccgggacctccaccctggaagtatacgatccc
CL_a        gtatggatctacggaggcggattctattccgggacctccaccctggaagtatacgatccc
AL_b        gtatggatctacggaggcggattctattccgggacctccaccctggaagtatacgatccc
LR1_a       gtatggatctacggaggcggattctattccgggacctccaccctggaagtatacgatccc
S1_a        gtatggatctacggaggcggattctattccgggacctccaccctggaagtatacgatccc
SS1_b       gtatggatctacggaggcggattctattccgggacctccaccctggaagtatacgatccc
            ************************************************************
```

```
LR2_b      cgcgtcatcgtctctgaggaaaacataatctttgtggccatgcagtaccgtgtggccagc
CL_b       cgcgtcatcgtctctgaggaaaacataatctttgtggccatgcagtaccgtgtggccagc
S3_b       cgcgtcatcgtctctgaggaaaacataatctttgtggccatgcagtaccgtgtggccagc
LR1_b      cgcgtcatcgtctctgaggaaaacataatctttgtggccatgcagtaccgtgtggccagc
LR2_a      cgcgtcatcgtctctgaggaaaacataatctttgtggccatgcagtaccgtgtggccagc
S2_a       cgcgtcatcgtctctgaggaaaacataatctttgtggccatgcagtaccgtgtggccagc
S4_b       cgcgtcatcgtctctgaggaaaacataatctttgtggccatgcagtaccgtgtggccagc
SS1_a      cgcgtcatcgtctctgaggaaaacataatctttgtggccatgcagtaccgtgtggccagc
S4_a       cgcgtcatcgtctctgaggaaaacataatctttgtggccatgcagtaccgtgtggccagc
S2_b       cgcgtcatcgtctctgaggaaaacataatctttgtggccatgcagtaccgtgtggccagc
BL_b       cgcgtcatcgtctctgaggaaaacataatctttgtggccatgcagtaccgtgtggccagc
BL_a       cgcgtcatcgtctctgaggaaaacataatctttgtggccatgcagtaccgtgtggccagc
AL_a       cgcgtcatcgtctctgaggaaaacataatctttgtggccatgcagtaccgtgtggccagc
S3_a       cgcgtcattgtctctgaggaaaacataatctttgtggccatgcagtaccgtgtggccagc
S1_b       cgcgtcatcgtctctgaggaaaacataatctttgtggccatgcagtaccgtgtggccagc
CL_a       cgcgtcatcgtctctgaggaaaacataatctttgtggccatgcagtaccgtgtggccagc
AL_b       cgcgtcatcgtctctgaggaaaacataatctttgtggccatgcagtaccgtgtggccagc
LR1_a      cgcgtcatcgtctctgaggaaaacataatctttgtggccatgcagtaccgtgtggccagc
S1_a       cgcgtcatcgtctctgaggaaaacataatctttgtggccatgcagtaccgtgtggccagc
SS1_b      cgcgtcatcgtctctgaggaaaacataatctttgtggccatgcagtaccgtgtggccagc
           ******** ***************************************************

LR2_b      ctgggattcctcttcttcgacaacgaggaagtccctggaaacgcgggactttacgatcaa
CL_b       ctgggattcctattcttcgacaacgaggaagtccctggaaacgcgggactttacgatcaa
S3_b       ctgggattcctattcttcgacaacgaggaagtccctggaaacgcgggactttacgatcaa
LR1_b      ctgggattcctcttcttcgacaacgaggaagtccctggaaacgcgggactttacgatcaa
LR2_a      ctgggattcctcttcttcgacaacgaggaagtccctggaaacgcgggactttacgatcaa
S2_a       ctgggattcctcttcttcgacaacgaggaagtccctggaaacgcgggactttacgatcaa
S4_b       ctgggattcctcttcttcgacaacgaggaagtccctggaaacgcgggactttacgatcaa
SS1_a      ctgggattcctcttcttcgacaacgaggaagtccctggaaacgcgggactttacgatcaa
S4_a       ctgggattcctcttcttcgacaacgaggaagtccctggaaacgcgggactttacgatcaa
S2_b       ctgggattcctcttcttcgacaacgaggaagtccctggaaacgcgggactttacgatcaa
BL_b       ctgggattcctcttcttcgacaacgaggaagtccctggaaacgcaggactttacgatcaa
BL_a       ctgggattcctcttcttcgacaacgaggaagtccctggaaacgcgggactttacgatcaa
AL_a       ctgggattcctcttcttcgacaacgaggaagtccctggaaacgcgggactttacgatcaa
S3_a       ctgggattcctcttcttcgacaacgaggaagtccctggaaacgcgggactttacgatcaa
S1_b       ctgggattcctcttcttcgacaacgaggaagtccctggaaacgcgggactttacgatcaa
CL_a       ctgggattcctcttcttcgacaacgaggaagtccctggaaacgcgggactttacgatcaa
AL_b       ctgggattcctcttcttcgacaacgaggaagtccctggaaacgcgggactttacgatcaa
LR1_a      ctgggattcctcttcttcgacaacgaggaagtccctggaaacgcgggactttacgatcaa
S1_a       ctgggattcctcttcttcgacaacgaggaagtccctggaaacgcgggactttacgatcaa
SS1_b      ctgggattcctcttcttcgacaacgaggaagtccctggaaacgcgggactttacgatcaa
           *********** ******************************** ***************

LR2_b      atgatggcgctgcagtgggtcaaggaaaacatcaaggagttcggaggggatcctgagagg
CL_b       atgatggcgctgcagtgggtcaaggaaaacatcaaggagttcggaggggatcctgagagg
S3_b       atgatggcgctgcagtgggtcaaggaaaacatcaaggagttcggaggggatcctgagagg
LR1_b      atgatggcgctgcagtgggtcaaggaaaacatcaaggagttcggaggggatcctgagagg
LR2_a      atgatggcgctgcagtgggtcaaggaaaacatcaaggagttcggaggggatcctgagagg
S2_a       atgatggcgctgcagtgggtcaaggaaaacatcaaggagttcggaggggatcctgagagg
S4_b       atgatggcgctgcaggggggtcaaggaaaacatcaaggagttcggaggggatcctgagagg
SS1_a      atgatggcgctgcagtgggtcaaggaaaacatcaaggagttcggaggggatcctgagagg
S4_a       atgatggcgctgcagtgggtcaaggaaaacatcaaggagttcggaggggatcctgagagg
S2_b       atgatggcgctgcagtgggtcaaggaaaacatcaaggagttcggaggggatcctgagagg
BL_b       atgatggcgctgcagtgggtcaaggaaaacatcaaggagttcggaggggatcctgagagg
BL_a       atgatggcgctgcagtgggtcaaggaaaacatcaaggagttcggaggggatcctgagagg
AL_a       atgatggcgctgcagtgggtcaaggaaaacatcaaggagttcggaggggatcctgagagg
S3_a       atgatggcgctgcagtgggtcaaggaaaacatcaaggagttcggaggggatcctgagagg
S1_b       atgatggcgctgcagtgggtcaaggaaaacatcaaggagttcggaggggatcctgagagg
CL_a       atgatggcgctgcagtgggtcaaggaaaacatcaaggagttcggaggggatcctgagagg
AL_b       atgatggcgctgcagtgggtcaaggaaaacatcaaggagttcggaggggatcctgagagg
LR1_a      atgatggcgctgcagtgggtcaaggaaaacatcaaggagttcggaggggatcctgagagg
S1_a       atgatggcgctgcagtgggtcaaggaaaacatcaaggagttcggaggggatcctgagagg
SS1_b      atgatggcgctgcagtgggtcaaggaaaacatcaaggagttcggaggggatcctgagagg
           *************** ********************************************
```

```
LR2_b       attacaatctttggagagtctgccggagcctgctcagtggccctgcatctcctttctccc
CL_b        attacaatctttggagagtctgccggagcctgctcagtggccctgcatctcctttctccc
S3_b        attacaatctttggagagtctgccggagcctgctcagtggccctgcatctcctttctccc
LR1_b       attacaatctttggagagtctgccggagcctgctcagtggccctgcatctcctttctccc
LR2_a       attacaatctttggagagtctgccggagcctgctcagtggccctgcatctcctttctccc
S2_a        attacaatctttggagagtctgccggagcctgctcagtggccctgcatctcctttctccc
S4_b        attacaatctttggagagtctgccggagcctgctcagtggccctgcatctcctttctccc
SS1_a       attacaatctttggagagtctgccggagcctgctcagtggccctgcatctcctttctccc
S4_a        attacaatctttggagagtctgccggagcctgctcagtggccctgcatctcctttctccc
S2_b        attacaatctttggagagtctgccggagcctgctcagtggccctgcatctcctttctccc
BL_b        attacaatctttggagagtctgccggagcctgctcagtggccctgcatctcctttctccc
BL_a        attacaatctttggagagtctgccggagcctgctcagtggccctgcatctcctttctccc
AL_a        attacaatctttggagagtctgccggagcctgctcagtggccctgcatctcctttctccc
S3_a        attacaatctttggagagtctgccggagcctgctcagtggccctgcatctcctttctccc
S1_b        attacaatctttggagagtctgccggagcctgctcagtggccctgcatctcctttctccc
CL_a        attacaatctttggagagtctgccggagcctgctcagtggccctgcatctcctttctccc
AL_b        attacaatctttggagagtctgccggagcctgctcagtggccctgcatctcctttctccc
LR1_a       attacaatctttggagagtctgccggagcctgctcagtggccctgcatctcctttctccc
S1_a        attacaatctttggagagtctgccggagcctgctcagtggccctgcatctcctttctccc
SS1_b       attacaatctttggagagtctgccggagcctgctcagtggccctgcatctcctttctccc
            ************************************************************

LR2_b       ctctcacgaaacctcttctcccaggccatcatgcaaagctcctccgcactcgttccctgg
CL_b        ctctcacgaaacctcttctcccaggccatcatgcaaagctcctccgcactcgttccctgg
S3_b        ctctcacgaaacctcttctcccaggccatcatgcaaagctcctccgcactcgttccctgg
LR1_b       ctctcacgaaacctattctcccaggccatcatgcaaagctcctccgcactcgttccctgg
LR2_a       ctctcacgaaacctcttctcccaggccatcatgcaaagctcctccgcactcgttccctgg
S2_a        ctctcacgaaacctcttctcccaggccatcatgcaaagctcctccgcactcgttccctgg
S4_b        ctctcacgaaacctcttctcccaggccatcatgcaaagctcctctgcactcgttccctgg
SS1_a       ctctcacgaaacctcttctcccaggccatcatgcaaagctcctccgcactcgttccctgg
S4_a        ctctcacgaaacctcttctcccaggccatcatgcaaagctcctctgcactcgttccctgg
S2_b        ctctcacgaaacctcttctcacaggccatcatgcaaagctcctccgcactcgttccctgg
BL_b        ctctcacgaaacctcttctcccaggccatcatgcaaagctcctccgcactcgttccctgg
BL_a        ctctcacgaaacctcttctcccaggccatcatgcaaagctcctccgcactcgttccctgg
AL_a        ctctcacgaaacctcttctcccaggccatcatgcaaagctcctccgcactcgttccctgg
S3_a        ctctcacgaaacctcttctcccaggccatcatgcaaagctcctccgcactcgttccctgg
S1_b        ctctcacgaaacctcttctcccaggccatcatgcaaagctcctccgcactcgttccctgg
CL_a        ctctcacgaaacctcttctcccaggccatcatgcaaagctcctccgcactcgttccctgg
AL_b        ctctcacgaaacctcttctcccaggccatcatgcaaagctcctccgcactcgttccctgg
LR1_a       ctctcacgaaacctcttctcccaggccatcatgcaaagctcctccgcactcgttccctgg
S1_a        ctctcacgaaacctcttctcccaggccatcatgcaaagctcctccgcactcgttccctgg
SS1_b       ctctcacgaaacctcttctcccaggccatcatgcaaagctcctccgcactcgttccctgg
            ************** ***** ********************** ****************

LR2_b       ggcgttatcaccaagaaggagagcatcatccgtgggcaccgcctcgctaaaatgatgggt
CL_b        ggcgttatcaccaagaaggagagcatcatccgtgggcaccgcctcgctaaaatgatgggt
S3_b        ggcgttatcaccaagaaggagagcatcatccgtgggcaccgcctcgctaaaatgatgggt
LR1_b       ggcgttatcaccaagaaggagagcatcatccgtgggcaccgcctcgctaaaatgatgggt
LR2_a       ggcgttatcaccaagaaggagagcatcatccgtgggcaccgcctcgctaaaatgatgggt
S2_a        ggcgttatcaccaagaaggagagcatcatccgtgggcaccgcctcgctaaaatgatgggt
S4_b        ggcgttatcaccaagaaggagagcatcatccgtgggcaccgcctcgctaaaatgatgggt
SS1_a       ggcgttatcaccaagaaggagagcatcatccgtgggcaccgcctcgctaaaatgatgggt
S4_a        ggcgttatcaccaagaaggagagcatcatccgtgggcaccgcctcgctaaaatgatgggt
S2_b        ggcgttatcaccaagaaggagagcatcatccgtgggcaccgcctcgctaaaatgatgggt
BL_b        ggcgttatcaccaagaaggagagcatcatccgtgggcaccgcctcgctaaaatgatgggt
BL_a        ggcgttatcaccaagaaggagagcatcatccgtgggcaccgcctcgctaaaatgatgggt
AL_a        ggcgttatcaccaagaaggagagcatcatccgtgggcaccgcctcgctaaaatgatgggt
S3_a        ggcgttatcaccaagaaggagagcatcatccgtgggcaccgcctcgctaaaatgatgggt
S1_b        ggcgttatcaccaagaaggagagcatcatccgtgggcaccgcctcgctaaaatgatgggt
CL_a        ggcgttatcaccaagaaggagagcatcatccgtgggcaccgcctcgctaaaatgatgggt
AL_b        ggcgttatcaccaagaaggagagcatcatccgtgggcaccgcctcgctaaaatgatgggt
LR1_a       ggcgttatcaccaagaaggagagcatcatccgtgggcaccgcctcgctaaaatgatgggt
S1_a        ggcgttatcaccaagaaggagagcatcatccgtgggcaccgcctcgctaaaatgatgggt
SS1_b       ggcgttatcaccaagaaggagagcatcatccgtgggcaccgcctcgctaaaatgatgggt
            ************************************************************
```

```
LR2_b      tgtcctcatgaagaaaaggatacgaagcccatgattgagtgtttgcgtaatcagagcgcc
CL_b       tgtcctcatgaagaaaaggatacgaagcccatgattgagtgtttgcgtaatcagagcgcc
S3_b       tgtcctcatgaagaaaaggatacgaagcccatgattgagtgtttgcgtaatcagagcgcc
LR1_b      tgtcctcatgaagaaaaggatacgaagcccatgattgagtgtttgcgtaatcagagcgcc
LR2_a      tgtcctcatgaagaaaaggatacgaagcccatgattgagtgtttgcgtaatcagagcgcc
S2_a       tgtcctcatgaagaaaaggatacgaagcccatgattgagtgtttgcgtaatcagagcgcc
S4_b       tgtcctcatgaagaaaaggatacgaagcccatgattgagtgtttgcgtaatcagagcgcc
SS1_a      tgtcctcatgaagaaaaggatacgaagcccatgattgagtgtttgcgtaatcagagcgcc
S4_a       tgtcctcatgaagaaaaggatacgaagcccatgattgagtgtttgcgtaatcagagcgcc
S2_b       tgtcctcatgaagaaaaggatacgaagcccatgattgagtgtttgcgtaatcagagcgcc
BL_b       tgtcctcatgaagaaaaggatacgaagcccatgattgagtgtttgcgtaatcagagcgcc
BL_a       tgtcctcatgaagaaaaggatacgaagcccatgattgagtgtttgcgtaatcagagcgcc
AL_a       tgtcctcatgaagaaaaggatacgaagcccatgattgagtgtttgcgtaatcagagcgcc
S3_a       tgtcctcatgaagaaaaggatacgaagcccatgattgagtgtttgcgtaatcagagcgcc
S1_b       tgtcctcatgaagaaaaggatacgaagcccatgattgagtgtttgcgtaatcagagcgcc
CL_a       tgtcctcatgaagaaaaggatacgaagcccatgattgagtgtttgcgtaatcagagcgcc
AL_b       tgtcctcatgaagaaaaggatacgaagcccatgattgagtgtttgcgtaatcagagcgcc
LR1_a      tgtcctcatgaagaaaaggatacgaagcccatgattgagtgtttgcgtaatcagagcgcc
S1_a       tgtcctcatgaagaaaaggatacgaagcccatgattgagtgtttgcgtaatcagagcgcc
SS1_b      tgtcctcatgaagaaaaggatacgaagcccatgattgagtgtttgcgtaatcagagcgcc
           ************************************************************

LR2_b      acggacatggtcaaccaggagtggctggggatcatttccgggattgcagagttccccttc
CL_b       acggacatggtcaaccaggagtggctgggcatcatttccgggattgcagagttccccttc
S3_b       acggacatggtcaaccaggagtggctgggcatcatttccgggattgcagagttccccttc
LR1_b      acggacatggtcaaccaggagtggctgggcatcatttccgggattgcagaggtccccttc
LR2_a      acggacatggtcaaccaggagtggctgggcatcatttccgggattgcagaggtccccttc
S2_a       acggacatggtcaaccaggagtggctgggcatcatttccgggattgcagaggtccccttc
S4_b       acggacatggtcaaccaggaggggctgggcatcatttccgggattgcagagttccccttc
SS1_a      acggacatggtcaaccaggagtggctgggcatcatttccgggattgcagagttccccttc
S4_a       acggacatggtcaaccaggagtggctgggcatcatttccgggattgcagagttccccttc
S2_b       acggacatggtcaaccaggagtggctgggcatcatttccgggattgcagagttccccttc
BL_b       acggacatggtcaaccaggagtggctgggcatcatttccgggattgcagagttccccttc
BL_a       acggacatggtcaaccaggagtggctgggcatcatttccgggattgcagagttccccttc
AL_a       acggacatggtcaaccaggagtggctgggcattatttccgggattgcagagttccccttc
S3_a       acggacatggtcaaccaggagtggctgggcatcatttccgggattgcagagttccccttc
S1_b       acggacatggtcaaccaggagtggctgggcatcatttccgggattgcagagttccccttc
CL_a       acggacatggtcaaccaggagtggctgggcatcatttccgggattgcagagttccccttc
AL_b       acggacatggtcaaccaggagtggctgggcatcatttccgggattgcagagttccccttc
LR1_a      acggacatggtcaaccaggagtggctgggcatcatttccgggattgcagagttccccttc
S1_a       acggacatggtcaaccaggagtggctgggcatcatttccgggattgcagagttccccttc
SS1_b      acggacatggtcaaccaggagtggctgggcatcatttccgggattgcagagttccccttc
           ********************* ******* ** ****************** ********

LR2_b      gttccaattgtggacggtagtcttctggatgaaagccctggaaagtccctcagctccaaa
CL_b       gttccaattgtggacgggagtcttctggatgaaagccctggaaagtccctcagctccaaa
S3_b       gttccaattgtggacgggagtcttctggatgaaagccctggaaagtccctcagctccaaa
LR1_b      gttccaattgtggacgggagtcttctggatgaaagccctggaaagtccctcagctccaaa
LR2_a      gttccaattgtggacgggagtcttctggatgaaagccctggaaagtccctcagctccaaa
S2_a       gttccaattgtggacgggagtcttctggatgaaagccctggaaagtccctcagctccaaa
S4_b       gttccaattgtggacgggagtcttctggatgaaagccctggaaagtccctcagctccaaa
SS1_a      gttccaattgtggacgggagtcttctggatgaaagccctggaaagtccctcagctccaaa
S4_a       gttccaattgtggacgggagtcttctggatgaaagccctggaaagtccctcagctccaaa
S2_b       gttccaattgtggacgggagtcttctggatgaaagccctggaaagtccctcagctccaaa
BL_b       gttccaattgtggacgggagtcttctggatgaaagccctggaaagtccctcagctccaaa
BL_a       gttccaattgtggacgggagtcttctggatgaaagccctggaaagtccctcagctccaaa
AL_a       gttccaattgtggacgggagtcttctggatgaaagccctggaaagtccctcagctccaaa
S3_a       gttccaattgtggacgggagtcttctggatgaaagccctggaaagtccctcagctccaaa
S1_b       gttccaattgtggacgggagtcttctggatgaaagccctggaaagtccctcagctccaaa
CL_a       gttccaattgtggacgggagtcttctggatgaaagccctggaaagtccctcagctccaaa
AL_b       gttccaattgtggacgggagtcttctggatgaaagccctggaaagtccctcagctccaaa
LR1_a      gttccaattgtggacgggagtcttctggatgaaagccctggaaagtccctcagctccaaa
S1_a       gttccaattgtggacgggagtcttctggatgaaagccctggaaagtccctcagctccaaa
SS1_b      gttccaattgtggacgggagtcttctggatgaaagccctggaaagtccctcagctccaaa
           ***************** ******************************************
```

```
LR2_b       aactacaagaagacaaacattcttatcggggcaaacaaggaggaagggaactactttctg
CL_b        aactacaagaagacaaacattcttatcggggcaaacaaggaggaagggaactacttttttg
S3_b        aactacaagaagacaaacattcttatcggggcaaacaaggaggaagggaactactttctg
LR1_b       aactacaagaagacaaacattcttatcggggcaaacaaggaggaagggaactactttctg
LR2_a       aactacaagaagacaaacattcttatcggggcaaacaaggaggaagggaactactttttg
S2_a        aactacaagaagacaaacattcttatcggggcaaacaaggaggaagggaactactttctg
S4_b        aactacaagaagacaaacattcttatcggggcaaacaaggaggaagggaactactttctg
SS1_a       aactacaagaagacaaacattcttatcggggcaaacaaggaggaagggaactactttctg
S4_a        aactacaagaagacaaacattcttatcggggcaaacaaggaggaagggaactactttctg
S2_b        aactacaagaagacaaacattcttatcggggcaaacaaggaggaagggaactactttctg
BL_b        aactacaagaagacaaacattcttatcggggcaaacaaggaggaagggaactactttctg
BL_a        aactacaagaagacaaacattcttatcggggcaaacaaggaggaagggaactactttttg
AL_a        aactacaagaagacaaacattcttatcggggcaaacaaggaggaagggaactactttctg
S3_a        aactacaagaagacaaacattcttatcggggcaaacaaggaggaagggaactactttctg
S1_b        aactacaagaagacaaacattcttatcggggcaaacaaggaggaagggaactactttctg
CL_a        aactacaagaagacaaacattcttatcggggcaaacaaggaggaagggaactactttctg
AL_b        aactacaagaagacaaacattcttatcggggcaaacaaggaggaagggaactactttctg
LR1_a       aactacaagaagacaaacattcttatcggggcaaacaaggaggaagggaactactttctg
S1_a        aactacaagaagacaaacattcttatcggggcaaacaaggaggaagggaactactttctg
SS1_b       aactacaagaagacaaacattcttatcggggcaaacaaggaggaagggaactactttctg
            ********************************************************* **

LR2_b       ctctactatctcacagatctcttcaaaaacacagagagcgtctatgtggaccgggctgac
CL_b        ctctactatctcacagatctcttcaaaaacacagagagcgtctatgtggaccgggctgac
S3_b        ctctactatctcacagatctcttcaaaaacacagagagcgtctatgtggaccgggctgac
LR1_b       ctctactatctcacagatctcttcaaaaacacagagagcgtctatgtggaccgggctgac
LR2_a       ctctactatctcacagatctcttcaaaaacacagagagcgtctatgtggaccgggctgac
S2_a        ctctactatctcacagatctcttcaaaaacacagagagcgtctatgtggaccgggctgac
S4_b        ctctactatctcacagatctcttcaaaaacacagagagcgtctatgtggaccgggctgac
SS1_a       ctctactatctcacagatctcttcaaaaacacagagagcgtctatgtggaccgggctgac
S4_a        ctctactatctcacagatctcttcaaaaacacagagagcgtctatgtggaccgggctgac
S2_b        ctctactatctcacagatctcttcaaaaacacagagagcgtctatgtggaccgggctgac
BL_b        ctctactatctcacagatctcttcaaaaacacagagagcgtctatgtggaccgggctgac
BL_a        ctctactatctcacagatctcttcaaaaacacagagagcgtctatgtggaccgagctgac
AL_a        ctctactatctcacagatctcttcaaaaacacagagagcgtctatgtggaccgggctgac
S3_a        ctctactatctcacagatctcttcaaaaacacagagagcgtctatgtggaccgggctgac
S1_b        ctctactatctcacagatctcttcaaaaacacagagagcgtctatgtggaccgggctgac
CL_a        ctctactatctcacagatctcttcaaaaacacagagagcgtctatgtggaccgggctgac
AL_b        ctctactatctcacagatctcttcaaaaacacagagagcgtctatgtggaccgggctgac
LR1_a       ctctactatctcacagatctcttcaaaaacacagagagcgtctatgtggaccgggctgac
S1_a        ctctactatctcacagatctcttcaaaaacacagagagcgtctatgtggaccgggctgac
SS1_b       ctctactatctcacagatctcttcaaaaacacagagagcgtctatgtggaccgggctgac
            ***************************************************** ******

LR2_b       tttattcgaagtgtggcggagctaaaccactacgtgaacaaggtggggcgggaggccatt
CL_b        tttattcgaagtgtggcggagctaaaccactacgtgaacaaggtggggcgggaggccatt
S3_b        tttattcgaagtgtggcggagctaaaccactacgtgaacaaggtggggcgggaggccatt
LR1_b       tttattcgaagtgtggcggagctaaaccactacgtgaacaaggtggggcgggaggccatt
LR2_a       tttattcgaagtgtggcggagctaaaccactacgtgaacaaggtggggcgggaggccatt
S2_a        tttattcgaagtgtggcggagctaaaccactacgtgaacaaggtggggcgggaggccatt
S4_b        tttattcgaagtgtggcggagctaaaccactacgtgaacaaggtggggcgggaggccatt
SS1_a       tttattcgaagtgtggcggagctaaaccactacgtgaacaaggtggggcgggaggccatt
S4_a        tttattcgaagtgtggcggagctaaaccactacgtgaacaaggtggggcgggaggccatt
S2_b        tttattcgaagtgtggcggagctaaaccactacgtgaacaaggtggggcgggaggccatt
BL_b        tttattcgaagtgtggcggagctaaaccactacgtgaacaaggtggggcgggaggccatt
BL_a        tttattcgaagtgtggcggagctaaaccactacgtgaacaaggtggggcgggaggccatt
AL_a        tttattcgaagtgtggcggagctaaaccactacgtgaacaaggtggggcgggaggccatt
S3_a        tttattcgaagtgtggcggagctaaaccactacgtgaacaaggtggggcgggaggccatt
S1_b        tttattcgaagtgtggcggagctaaaccactacgtgaacaaggtggggcgggaggccatt
CL_a        tttattcgaagtgtggcggagctaaaccactacgtgaacaaggtggggcgggaggccatt
AL_b        tttattcgaagtgtggcggagctaaaccactacgtgaacaaggtggggcgggaggccatt
LR1_a       tttattcgaagtgtggcggagctaaaccactacgtgaacaaggtggggcgggaggccatt
S1_a        tttattcgaagtgtggcggagctaaaccactacgtgaacaaggtggggcgggaggccatt
SS1_b       tttattcgaagtgtggcggagctaaaccactacgtgaacaaggtggggcgggaggccatt
            ************************************************************
```

```
LR2_b        atatttgagtacacagactggctgaatcccaatgatgccatcaagaatcgggagtccgtg
CL_b         atatttgagtacacagactggctgaatcccaatgatgccatcaagaatcgggagtccgtg
S3_b         atatttgagtacacagactggctgaatcccaatgatgccatcaagaatcgggagtccgtg
LR1_b        atatttgagtacacagactggctgaatcccaatgatgccatcaaaaatcgggagtccgtg
LR2_a        atatttgagtacacagactggctgaatcccaatgatgccatcaaaaatcgggagtccgtg
S2_a         atatttgagtacacagactggctgaatcccaatgatgccatcaaaaatcgggagtccgtg
S4_b         atatttgagtacacagactggctgaatcccaatgatgccatcaaaaatcgggagtccgtg
SS1_a        atatttgagtacacagactggctgaatcccaatgatgccatcaaaaatcgggagtccgtg
S4_a         atatttgagtacacagactggctgaatcccaatgatgccatcaaaaatcgggagtccgtg
S2_b         atatttgagtacacagactggctgaatcccaatgatgccatcaaaaatcgggagtccgtg
BL_b         atatttgagtacacagactggctgaatcccaatgatgccatcaaaaatcgggagtccgtg
BL_a         atatttgagtacacagactggctgaatcccaatgatgccatcaaaaatcgggagtccgtg
AL_a         atatttgagtacacagactggctgaatcccaatgatgccatcaaaaatcgggagtccgtg
S3_a         atatttgagtacacagactggctgaatcccaatgatgccatcaaaaatcgggagtccgtg
S1_b         atatttgagtacacagactggctgaatcccaatgatgccatcaaaaatcgggagtccgtg
CL_a         atatttgagtacacagactggctgaatcccaatgatgccatcaaaaatcgggagtccgtg
AL_b         atatttgagtacacagactggctgaatcccaatgatgccatcaaaaatcgggagtccgtg
LR1_a        atatttgagtacacagactggctgaatcccaatgatgccatcaaaaatcgggagtccgtg
S1_a         atatttgagtacacagactggctgaatcccaatgatgccatcaaaaatcgggagtccgtg
SS1_b        atatttgagtacacagactggctgaatcccaatgatgccatcaaaaatcgggagtccgtg
             ******************************************** ***************

LR2_b        gatcgcatggttggggactatcagttcacttgccccactgcagactttgctcggatctac
CL_b         gatcgcatggttggggactatcagttcacttgccccactgcagactttgctcggatctac
S3_b         gatcgcatggttggggactatcagttcacttgccccactgcagactttgctcggatctac
LR1_b        gatcgcatggttggggactatcagttcacttgccccactgcagactttgctcggatctac
LR2_a        gatcgcatggttggggactatcagttcacttgccccactgcagactttgctcggatctac
S2_a         gatcgcatggttggggactatcagttcacttgccccactgcagactttgctcggatctac
S4_b         gatcgcatggttggggactatcagttcacttgccccactgcagactttgctcggatctac
SS1_a        gatcgcatggttggggactaccagttcacttgccccactgcagactttgctcggatctac
S4_a         gatcgcatggttggggactatcagttcacttgccccactgcagactttgctcggatctat
S2_b         gatcgcatggttggggactatcagttcacttgccccactgcagactttgctcggatctac
BL_b         gatcgcatggttggggactatcagttcacttgccccactgcagactttgctcggatctac
BL_a         gatcgcatggttggggactatcagttcacttgccccactgcagactttgctcggatctac
AL_a         gatcgtatggttggggactatcagttcacttgccccactgcagactttgctcggatctac
S3_a         gatcgcatggttggggactatcagttcacttgccccactgcagactttgctcggatctac
S1_b         gatcgcatggttggggactatcagttcacttgccccactgcagactttgctcggatctat
CL_a         gatcgcatggttggggactatcagttcacttgccccactgcagactttgctcggatctac
AL_b         gatcgcatggttggggactatcagttcacttgccccactgcagactttgctcggatctac
LR1_a        gatcgcatggttggggactatcagttcacttgccccactgcagactttgctcggatctac
S1_a         gatcgcatggttggggactatcagttcacttgccccactgcagactttgctcggatctac
SS1_b        gatcgcatggttggggactatcagttcacttgccccactgcagactttgctcggatctac
             ***** ************** ************************************** 

LR2_b        gccagcacgggcaacaatgtctacatgtactacttcacggagcgctcctccatgaaccca
CL_b         gccagcacgggcaacaatgtctacatgtactacttcacggagcgctcctccatgaaccca
S3_b         gccagcacgggcaacaatgtctacatgtactacttcacggagcgctcctccatgaaccca
LR1_b        gccagcacgggcaacaatgtctacatgtactacttcacggagcggtcctccatgaaccca
LR2_a        gccagcacgggcaacaatgtctacatgtactacttcacggagcggtcctccatgaaccca
S2_a         gccagcacgggcaacaatgtctacatgtactacttcacggagcggtcctccatgaaccca
S4_b         gccagcacgggcaacaatgtctacatgtactacttcacagagcggtcctccatgaaccca
SS1_a        gccagcacgggcaacaatgtctacatgtactacttcacggagcggtcctccatgaaccca
S4_a         gccagcacgggcaacaatgtctacatgtactacttcacggagcggtcctccatgaaccca
S2_b         gccagcacgggcaacaatgtctacatgtactacttcacggagcggtcctccatgaaccca
BL_b         gccagcacgggcaacaatgtctacatgtactacttcacggagcggtcctccatgaaccca
BL_a         gccagcacgggcaacaatgtctacatgtactacttcacggagcggtcctccatgaaccca
AL_a         gccagcacgggcaacaatgtctacatgtactacttcacggagcggtcctccatgaaccca
S3_a         gccagcacgggcaacaatgtctacatgtactacttcacggagcggtcctccatgaaccca
S1_b         gccagcacgggcaacaatgtctacatgtactacttcacggagcggtcctccatgaaccca
CL_a         gccagcacgggcaacaatgtctacatgtactacttcacggagcggtcctccatgaaccca
AL_b         gccagcacgggcaacaatgtctacatgtactacttcacggagcggtcctccatgaaccca
LR1_a        gccagcacgggcaacaatgtctacatgtactacttcacggagcggtcctccatgaaccca
S1_a         gccagcacgggcaacaatgtctacatgtactacttcacggagcggtcctccatgaaccca
SS1_b        gccagcacgggcaacaatgtctacatgtactacttcacggagcggtcctccatgaaccca
             ************************************** ***** ***************
```

```
LR2_b      tggccaacctggtccggagtgctccacggagacgaaattgcattcatctttggcgagcca
CL_b       tggccaacctggtccggagtgctccacggagacgaaattgcattcatctttggcgagcca
S3_b       tggccaacctggtccggagtgctccacggagacgaaattgcattcatctttggcgagcca
LR1_b      tggccaacctggtctggagtgctccacggagacgaaattgcattcatctttggcgagcca
LR2_a      tggccaacctggtctggagtgctccacggagacgaaattgcattcatctttggcgagcca
S2_a       tggccaacctggtctggagtgctccacggagacgaaattgcattcatctttggcgagcca
S4_b       tggccaacctggtctggagtgctccacggagacgaaattgcattcatctttggcgagcca
SS1_a      tggccaacctggtctggagtgctccacggagacgaaattgcattcatctttggcgagcca
S4_a       tggccaacctggtctggagtgctccacggagacgaaattgcattcatctttggcgagcca
S2_b       tggccaacctggtctggagtgctccacggagacgaaattgcattcatctttggcgagcca
BL_b       tggccaacctggtctggagtgctccacggagacgaaattgcattcatctttggcgagcca
BL_a       tggccaacctggtctggagtgctccacggagacgaaattgcattcatctttggcgagcca
AL_a       tggccaacctggtctggagtgctccacggagacgaaattgcattcatctttggcgagcca
S3_a       tggccaacctggtctggagtgctccacggagacgaaattgcattcatctttggcgagcca
S1_b       tggccaacctggtctggagtgctccacggagacgaaattgcattcatctttggcgagcca
CL_a       tggccaacctggtctggagtgctccacggagacgaaattgcattcatctttggcgagcca
AL_b       tggccaacctggtctggagtgctccacggagacgaaattgcattcatctttggcgagcca
LR1_a      tggccaacctggtctggagtgctccacggagacgaaattgcattcatctttggcgagcca
S1_a       tggccaacctggtctggagtgctccacggagacgaaattgcattcatctttggcgagcca
SS1_b      tggccaacctggtctggagtgctccacggagacgaaattgcattcatctttggcgagcca
           ************** *********************************************

LR2_b      ctcaataagtcaaagaactatgacaagtcagaaattgcactctccaagaggatgatggga
CL_b       ctcaataagtcaaagaactatgacaagtcagaaattgcactctccaagaggatgatggga
S3_b       ctcaataagtcaaagaactatgacaagtcagaaattgcactctccaagaggatgatggga
LR1_b      ctcaataagtcaaagaactatgacaagtcagaaattgcactctccaagaggatgatggga
LR2_a      ctcaataagtcaaagaactatgacaagtcagaaattgcactctccaagaggatgatggga
S2_a       ctcaataagtcaaagaactatgacaagtcagaaattgcactctccaagaggatgatggga
S4_b       ctcaataagtcaaagaactatgacaagtcagaaattgcactctccaagaggatgatggga
SS1_a      ctcaataagtcaaagaactatgacaagtcagaaattgcactctccaagaggatgatggga
S4_a       ctcaataagtcaaagaactatgacaagtcagaaattgcactctccaagaggatgatggga
S2_b       ctcaataagtcaaagaactatgacaagtcagaaattgcactctccaagaggatgatggga
BL_b       ctcaataagtcaaagaactatgacaagtcagaaattgcactctccaagaggatgatggga
BL_a       ctcaataagtcaaagaactatgacaagtcagaaattgcactctccaagaggatgatggga
AL_a       ctcaataagtcaaagaactatgacaagtcagaaattgcactctccaagaggatgatggga
S3_a       ctcaataagtcaaagaactatgacaagtcagaaattgcactctccaagaggatgatggga
S1_b       ctcaataagtcaaagaactatgacaagtcagaaattgcactctccaagaggatgatggga
CL_a       ctcaataagtcaaagaactatgacaagtcagaaattgcactctccaagaggatgatggga
AL_b       ctcaataagtcaaagaactatgacaagtcagaaattgcactctccaagaggatgatggga
LR1_a      ctcaataagtcaaagaactatgacaagtcagaaattgcactctccaagaggatgatggga
S1_a       ctcaataagtcaaagaactatgacaagtcagaaattgcactctccaagaggatgatggga
SS1_b      ctcaataagtcaaagaactatgacaagtcagaaattgcactctccaagaggatgatggga
           ************************************************************

LR2_b      tactggactaactttgccaaaactggaaatccaagtctctccgctgatggaacttggacg
CL_b       tactggactaactttgccaaaactggaaatccaagtctctccgctgatggaacttggacg
S3_b       tactggactaactttgccaaaactggaaatccaagtctctccgctgatggaacttggacg
LR1_b      tactggactaactttgccaaaactggaaatccaagtctctccgctgatggaacttggacg
LR2_a      tactggactaactttgccaaaactggaaatccaagtctctccgctgatggaacttggacg
S2_a       tactggactaactttgccaaaactggaaatccaagtctctccgctgatggaacttggacg
S4_b       tactggactaactttgccaaaactggaaatccaagtctctccgctgatggaacttggacg
SS1_a      tactggactaactttgccaaaactggaaatccaagtctctccgctgatggaacttggacg
S4_a       tactggactaactttgccaaaactggaaatccaagtctctccgctgatggaacttggacg
S2_b       tactggactaactttgccaaaactggaaatccaagtctctccgctgatggaacttggacg
BL_b       tactggactaactttgccaaaactggaaatccaagtctctccgctgatggaacttggacg
BL_a       tactggactaactttgccaaaactggaaatccaagtctctccgctgatggaacttggacg
AL_a       tactggactaactttgccaaaactggaaatccaagtctctccgctgatggaacttggacg
S3_a       tactggactaactttgccaaaactggaaatccaagtctctccgctgatggaacttggacg
S1_b       tactggactaactttgccaaaactggaaatccaagtctctccgctgatggaacttggacg
CL_a       tactggactaactttgccaaaactggaaatccaagtctctccgctgatggaacttggacg
AL_b       tactggactaactttgccaaaactggaaatccaagtctctccgctgatggaacttggacg
LR1_a      tactggactaactttgccaaaactggaaatccaagtctctccgctgatggaacttggacg
S1_a       tactggactaactttgccaaaactggaaatccaagtctctccgctgatggaacttggacg
SS1_b      tactggactaactttgccaaaactggaaatccaagtctctccgctgatggaacttggacg
           ************************************************************
```

```
LR2_b      gcaaactactggcctcttcacacacccactaaacaggaggtacttgaactaaacgccaat
CL_b       gcaaactactggcctcttcacacacccactaaacaggaggtacttgaactaaacgccaat
S3_b       gcaaactactggcctcttcacacacccactaaacaggaggtacttgaactaaacgccaat
LR1_b      gcaaactactggcctcttcacacacccactaaacaggaggtacttgaactaaacgccaat
LR2_a      gcaaactactggcctcttcacacacccactaaacaggaggtacttgaactaaacgccaat
S2_a       gcaaactactggcctcttcacacacccactaaacaggaggtacttgaactaaacgccaat
S4_b       gcaaactactggcctcttcacacacccactaaacaggaggtacttgaactaaacgccaat
SS1_a      gcaaactactggcctcttcacacacccactaaacaggaggtacttgaactaaacgccaat
S4_a       gcaaactactggcctcttcacacacccactaaacaggaggtacttgaactaaacgccaat
S2_b       gcaaactactggcctcttcacacacccactaaacaggaggtacttgaactaaacgccaat
BL_b       gcaaactactggcctcttcacacacccactaaacaggaggtacttgaactaaacgccaat
BL_a       gcaaactactggcctcttcacacacccactaaacaggaggtacttgaactaaacgccaat
AL_a       gcaaactactggcctcttcacacacccactaaacaggaggtacttgaactaaacgccaat
S3_a       gcaaactactggcctcttcacacacccactaaacaggaggtacttgaactaaacgccaat
S1_b       gcaaactactggcctcttcacacacccactaaacaggaggtacttgaactaaacgccaat
CL_a       gcaaactactggcctcttcacacacccactaaacaggaggtacttgaactaaacgccaat
AL_b       gcaaactactggcctcttcacacacccactaaacaggaggtacttgaactaaacgccaat
LR1_a      gcaaactactggcctcttcacacacccactaaacaggaggtacttgaactaaacgccaat
S1_a       gcaaactactggcctcttcacacacccactaaacaggaggtacttgaactaaacgccaat
SS1_b      gcaaactactggcctcttcacacacccactaaacaggaggtacttgaactaaacgccaat
           ************************************************************

LR2_b      tattctcgagttcttgagggtcttcgagtgaacaaatgtgccttttggaaaagatatcta
CL_b       tattctcgagttcttgagggtcttcgagtgaacaaatgtgccttttggaaaagatatcta
S3_b       tattctcgagttcttgagggtcttcgagtgaacaaatgtgccttttggaaaagatatcta
LR1_b      tattctcgagttcttgagggtcttcgagtgaacaaatgtgccttttggaaaagatatcta
LR2_a      tattctcgagttcttgagggtcttcgagtgaacaaatgtgccttttggaaaagatatcta
S2_a       tattctcgagttcttgagggtcttcgagtgaacaaatgtgccttttggaaaagatatcta
S4_b       tattctcgagttcttgagggtcttcgagtgaacaaatgtgccttttggaaaagatatcta
SS1_a      tattctcgagttcttgagggtcttcgagtgaacaaatgtgccttttggaaaagatatcta
S4_a       tattctcgagttcttgagggtcttcgagtgaacaaatgtgccttttggaaaagatatcta
S2_b       tattctcgagttcttgagggtcttcgagtgaacaaatgtgccttttggaaaagatatcta
BL_b       tattctcgagttcttgagggtcttcgagtgaacaaatgtgccttttggaaaagatatcta
BL_a       tattctcgagttcttgagggtcttcgagtgaacaaatgtgccttttggaaaagatatcta
AL_a       tattctcgagttcttgagggtcttcgagtgaacaaatgtgccttttggaaaagatatcta
S3_a       tattctcgagttcttgagggtcttcgagtgaacaaatgtgccttttggaaaagatatcta
S1_b       tattctcgagttcttgagggtcttcgagtgaacaaatgtgccttttggaaaagatatcta
CL_a       tattctcgagttcttgagggtcttcgagtgaacaaatgtgccttttggaaaagatatcta
AL_b       tattctcgagttcttgagggtcttcgagtgaacaaatgtgccttttggaaaagatatcta
LR1_a      tattctcgagttcttgagggtcttcgagtgaacaaatgtgccttttggaaaagatatcta
S1_a       tattctcgagttcttgagggtcttcgagtgaacaaatgtgccttttggaaaagatatcta
SS1_b      tattctcgagttcttgagggtcttcgagtgaacaaatgtgccttttggaaaagatatcta
           ************************************************************

LR2_b      cctaaacttttatcattgacatctaacaatggagaccatcagtgtacgagttcctccagt
CL_b       cctaaacttttatcattgacatctaacaatggagaccatcagtgtacgagttcctccagt
S3_b       cctaaacttttatcattgacatctaacaatggagaccatcagtgtacgagttcctccagt
LR1_b      cctaaacttttatcattgacatctaacaatggagaccatcagtgtacgagttcctccagt
LR2_a      cctaaacttttatcattgacatctaacaatggagaccatcagtgtacgagttcctccagt
S2_a       cctaaacttttatcattgacatctaacaatggagaccatcagtgtacgagttcctccagt
S4_b       cctaaacttttatcattgacatctaacaatggagaccatcagtgtacgagttcctccagt
SS1_a      cctaaacttttatcattgacatctaacaatggagaccatcagtgtacgagttcctccagt
S4_a       cctaaacttttatcattgacatctaacaatggagaccatcagtgtacgagttcctccagt
S2_b       cctaaacttttatcattgacatctaacaatggagaccatcagtgtacgagttcctccagt
BL_b       cctaagcttttatcattgacatctaacaatggagaccatcagtgtacgagttcctccagt
BL_a       cctaaacttttatcattgacatctaacaatggagaccatcagtgtacgagttcctccagt
AL_a       cctaaacttttatcattgacatctaacaatggagaccatcagtgtacgagttcctccagt
S3_a       cctaaacttttatcattgacatctaacaatggagaccatcagtgtacgagttcctccagt
S1_b       cctaaacttttatcattgacatctaacaatggagaccatcagtgtacgagttcctccagt
CL_a       cctaaacttttatcattgacatctaacaatggagaccatcagtgtacgagttcctccagt
AL_b       cctaaacttttatcattgacatctaacaatggagaccatcagtgtacgagttcctccagt
LR1_a      cctaaacttttatcattgacatctaacaatggagaccatcagtgtacgagttcctccagt
S1_a       cctaaacttttatcattgacatctaacaatggagaccatcagtgtacgagttcctccagt
SS1_b      cctaaacttttatcattgacatctaacaatggagaccatcagtgtacgagttcctccagt
           ***** ******************************************************
```

```
LR2_b       tcctcctcaacatctccgtcatgctgcaaaggtggatcatgctgtggtggaagcggagag
CL_b        tcctcctcaacatctccgtcatgctgcaaaggtggatcatgctgtggtggaagcggagag
S3_b        tcctcctcaacatctccgtcatgctgcaaaggtggatcatgctgtggtggaagcggagag
LR1_b       tcctcctcaacatctccgtcatgctgcaaaggtggatcatgctgtggtggaagcggagag
LR2_a       tcctcctcaacatctccgtcatgctgcaaaggtggatcatgctgtggtggaagcggagag
S2_a        tcctcctcaacatctccgtcatgctgcaaaggtggatcatgctgtggtggaagcggagag
S4_b        tcctcctcaacatctccgtcatgctgcaaaggtggatcatgctgtggtggaagcggagag
SS1_a       tcctcctcaacatctccgtcatgctgcaaaggtggatcatgctgtggtggaagcggagag
S4_a        tcctcctcaacatctccgtcatgctgcaaaggtggatcatgctgtggtggaagcggagag
S2_b        tcctcctcaacatctccgtcatgctgcaaaggtggatcatgctgtggtggaagcggagag
BL_b        tcctcctcaacatctccgtcatgctgcaaaggtggatcatgctgtggtggaagcggagag
BL_a        tcctcctcaacatctccgtcatgctgcaaaggtggatcatgctgtggtggaagcggagag
AL_a        tcctcctcaacatctccgtcatgctgcaaaggtggatcatgctgtggtggaagcggagag
S3_a        tcctcctcaacatctccgtcatgctgcaaaggtggatcatgctgtggtggaagcggagag
S1_b        tcctcctcaacatctccgtcatgctgcaaaggtggatcatgctgtggtggaagcggagag
CL_a        tcctcctcaacatctccgtcatgctgcaaaggtggatcatgctgtggtggaagcggagag
AL_b        tcctcctcaacatctccgtcatgctgcaaaggtggatcatgctgtggtggaagcggagag
LR1_a       tcctcctcaacatctccgtcatgctgcaaaggtggatcatgctgtggtggaagcggagag
S1_a        tcctcctcaacatctccgtcatgctgcaaaggtggatcatgctgtggtggaagcggagag
SS1_b       tcctcctcaacatctccgtcatgctgcaaaggtggatcatgctgtggtggaagcggagag
            ************************************************************

LR2_b       attccgggatcttcattcctcttaatcatcttcacttccacgctggtgcatctattttca
CL_b        attccgggatcttcattcctcttaatcatcttcacttccacgctggtgcatctattttca
S3_b        attccgggatcttcattcctcttaatcatcttcacttccacgctggtgcatctattttca
LR1_b       attccgggatcttcattcctcttaatcatcttcacttccacgctggtgcatctattttca
LR2_a       attccgggatcttcattcctcttaatcatcttcacttccacgctggtgcatctattttca
S2_a        attccgggatcttcattcctcttaatcatcttcacttccacgctggtgcatctattttca
S4_b        attccgggatcttcattcctcttaatcatcttcacttccacgctggtgcatctattttca
SS1_a       attccgggatcttcattcctcttaatcatcttcacttccacgctggtgcatctattttca
S4_a        attccgggatcttcattcctcttaatcatcttcacttccacgctggtgcatctattttca
S2_b        attccgggatcttcattcctcttaatcatcttcacttccacgctggtgcatctattttca
BL_b        attccgggatcttcattcctcttaatcatcttcacttccacgctggtgcatctattttca
BL_a        attccgggatcttcattcctcttaatcatcttcacttccacgctggtgcatctattttca
AL_a        attccgggatcttcattcctcttaatcatcttcacttccacgctggtgcatctattttca
S3_a        attccgggatcttcattcctcttaatcatcttcacttccacgctggtgcatctattttca
S1_b        attccgggatcttcattcctcttaatcatcttcacttccacgctggtgcatctattttca
CL_a        attccgggatcttcattcctcttaatcatcttcacttccacgctggtgcatctattttca
AL_b        attccgggatcttcattcctcttaatcatcttcacttccacgctggtgcatctattttca
LR1_a       attccgggatcttcattcctcttaatcatcttcacttccacgctggtgcatctattttca
S1_a        attccgggatcttcattcctcttaatcatcttcacttccacgctggtgcatctattttca
SS1_b       attccgggatcttcattcctcttaatcatcttcacttccacgctggtgcatctattttca
            ************************************************************

LR2_b       atctacaaacttagcataattaactactcaaataataataataatatttatacgaatcaa
CL_b        atctacaaacttagcataattaactactcaaataataataataatatttatacgaatcaa
S3_b        atctacaaacttagcataattaactactcaaataataataataatatttatacgaatcaa
LR1_b       atctacaaacttagcataattaactactcaaataataataataatatt------------
LR2_a       atctacaaacttagcataattaactactcaaataataataataatatttatacgaatcaa
S2_a        atctacaaacttagcataattaactactcaaataataataataatatttatacgaatcaa
S4_b        atctacaaacttagcataattaactactcaaataataataataatatttatacgaatcaa
SS1_a       atctacaaacttagcataattaactactcaaataataataataatatttatacgaatcaa
S4_a        atctacaaacttagcataattaactactcaaataataataataatatttatacgaatcaa
S2_b        atctacaaacttagcataattaactactcaaataataataataatatttatacgaatcaa
BL_b        atctacaaacttagcataattaactactcaaataataataataatatttatacgaatcaa
BL_a        atctacaaacttagcataattaactactcaaataataataataatatttatacgaatcaa
AL_a        atctacaaacttagcataattaactactcaaataataataataatatttatacgaatcaa
S3_a        atctacaaacttagcataattaactactcaaataataataataatatttatacgaatcaa
S1_b        atctacaaacttagcataattaactactcaaataataataataatatttatacgaatcaa
CL_a        atctacaaacttagcataattaactactcaaataataataataatatttatacgaatcaa
AL_b        atctacaaacttagcataattaactactcaaataataataataatatttatacgaatcaa
LR1_a       atctacaaacttagcataattaactactcaaataataataataatatt------------
S1_a        atctacaaacttagcataattaactactcaaataataataataatatttatacgaatcaa
SS1_b       atctacaaacttagcataattaactactcaaataataataataatatttatacgaatcaa
            ************************************************
```

```
LR2_b      aataataatggaaagctgtga
CL_b       aataataatggaaagctgtga
S3_b       aataataatggaaagctgtga
LR1_b      ---------------------
LR2_a      aataataatggaaagctgtga
S2_a       aataataatggaaagctgtga
S4_b       aataataatggaaagctgtga
SS1_a      aataataatggaaagctgtga
S4_a       aataataatggaaagctgtga
S2_b       aataataatggaaagctgtga
BL_b       aataataatggaaagctgtga
BL_a       aataataatggaaagctgtga
AL_a       aataataatggaaagctgtga
S3_a       aataataatggaaagctgtga
S1_b       aataataatggaaagctgtga
CL_a       aataataatggaaagctgtga
AL_b       aataataatggaaagctgtga
LR1_a      ---------------------
S1_a       aataataatggaaagctgtga
SS1_b      aataataatggaaagctgtga
```
