# Supplementary material for: Caligus rogercresseyi acetylcholinesterase types and variants: a potential marker for organophosphate resistance
Source: Parasit Vectors. 2018 Oct 30;11:570. doi: 10.1186/s13071-018-3151-7 (PMC6208076; doi:10.1186/s13071-018-3151-7)
Supplement: Supplementary file 9 — Alignment of six vertebrate butyrylcholinesterases (BChE), Caligus rogercresseyi AChE1a V318 variant (M) and Caligus rogercresseyi AChE1b. Figure showing a fragment of the alignment containing the amino acid corresponding to the position F290 (Torpedo californica AChE numbering) (highlighted). (PDF 165 kb) [file 13071_2018_3151_MOESM9_ESM.pdf]

Additional file 9. Alignment of six vertebrate butyrylcholinesterases (BChE), *Caligus rogercresseyi* AChE1a V318 variant (M) and *Caligus rogercresseyi* AChE1b. Figure showing a fragment of the alignment containing the amino acid corresponding to the position F290 (*T. californica* AChE numbering) (highlighted). UniprotKB database entry names: *Bos taurus* BChE: P32749|CHLE_BOVIN (B_taurus_BChE); *Felis catus* BChE: O62760|CHLE_FELCA (F_catus_BChE); *Gallus gallus* BChE: Q90ZK8|Q90ZK8_CHICK (G_gallus_BChE); *Homo sapiens* BChE: P06276|CHLE_HUMAN (H_sapiens_BChE); *Mus musculus* BChE: Q03311|CHLE_MOUSE (M_musculus_BChE); *Oryctolagus cuniculus* BChE: P21927|CHLE_RABIT (O_cuniculus_BChE).

```
Gallus_gallus_BChE                QDKDPKDILENEVYVVKYFSLLHIYFCPTVDGDFLADMPEALIKNGIFKQTQVLVGVNKD    352
Mus_musculus_BChE                 RSKDPQEILRNERFVLPSDSILSINFGPTVDGDFLTDMPHTLLQLGKVKKAQILVGVNKD    353
Felis_catus_BChE                  RNKDPQEILLNELLVVPSDTLLSVNFGPVVDGDFLTDMPDTLLQLGQFKKTQILVGVNKD    352
Bos_taurus_BChE                   RNKDPQEILRHEVFVVPYGTLLSVNFGPTVDGDFLTDMPDTLLQLGQFKKTQILVGVNKD    352
Homo_sapiens_BChE                 RNKDPQEILLNEAFVVPYGTPLSVNFGPTVDGDFLTDMPDILLELGQFKKTQILVGVNKD    352
Oryctolagus_cuniculus_BChE        RNKDAQEILLNEVFVVPFDSLLSVNFGPTVDGDFLTDMPDTLLQLGQLKKTQILVGVNKD    331
Caligus_rogercresseyi_AChE1a_M    RNQSATDMVNQE-WLGIISGIAEVPFVPIVDGSLLDESPGKSLSSKNYKKTNILIGANKE    354
Caligus_rogercresseyi_AChE1b      RGLKATQIMYLE-WESPRLRIMDIPFVPVLDGIFIPDNPREALHLGGYKKVNILIGTNED    317
                                  :. .  :::  *           : * * :** :: : *   :     *:.::*:*.*::
```
